# Identification of Gene Regulatory Network for Nitrogen-Promoted Tiller Regrowth in Perennial Rice

**DOI:** 10.1186/s12284-026-00901-z

**Published:** 2026-04-28

**Authors:** Yufan Lu, Guangfu Huang, Ziran Qi, Yujiao Zhang, Fang Liu, Lin Shao, Shilai Zhang, Fengyi Hu, Gui Jie Lei

**Affiliations:** 1 State Key Laboratory for Vegetation Structure, Key Laboratory of Biology and Germplasm Innovation of Perennial Rice, Center of Innovation for Perennial Rice Technology in Yunnan, New Cornerstone Science Laboratory, School of Agriculture, Yunnan University, Kunming, 650500 China; 2 Institute of International Rivers and Eco-Security, Yunnan University, Kunming, 650500 China

**Keywords:** Perennial rice, *Oryza longistaminata*, Nitrogen, Mineral nutrient, Auxin, Tiller bud, Rhizome

## Abstract

Perennial rice, which has shortened rhizomes derived from *Oryza longistaminata*, regenerates tillers from shoot basal regions after harvest, enabling multi-year perenniality. This crop represents an innovative approach to enhance global food security and promote sustainable agricultural development. However, the mechanisms regulating tiller regrowth in perennial rice remain poorly understood. Our study demonstrates that nitrogen (N) fertilizer application more effectively promotes tiller bud regrowth in perennial rice compared to annual rice. RNA-seq analysis identified 1038 differentially expressed genes (DEGs) in the shoot basal regions between perennial and annual rice after N application. Weighted Gene Co-expression Network Analysis (WGCNA) hierarchically clustered these DEGs into seven modules. The yellow module exhibited a significant positive correlation with tiller bud growth. Five core genes, *OsCCA1*, *OsRDD1*, *OsRDD2* and *OsMTG3* from the yellow module and *OsGSNOR* from the brown module involved in N or magnesium (Mg) utilization, exhibited significantly increased expression in perennial rice. Knockout of *OsCCA1* inhibited N-promoted tiller bud growth in perennial rice. OsCCA1 may regulate tiller bud growth by regulating hundreds of downstream genes based on the integration of RNA-seq data and the reported DAP-seq data. Additionally, perennial rice showed reduced indole-3-acetic acid (IAA) level and downregulated expression of auxin signaling response genes in shoot basal regions. Furthermore, IAA negatively regulated the expression of the five core genes and inhibited tiller growth in perennial rice. Our findings suggest that perennial rice efficiently utilizes N to promote tiller regrowth, potentially through regulating genes associated with IAA metabolism, auxin signaling, nutrient utilization, and plant growth.

**Supplementary Information:**

The online version contains supplementary material available at 10.1186/s12284-026-00901-z.

## Background

The rhizome, a horizontal underground stem capable of upward growth to form aerial stems or tillers, is a defining feature of many perennial herbs (Paterson et al. 1995; Hu et al. 2003; Zhang et al. 2022). Rhizome also functions as a critical energy storage organ that facilitates regrowth and survival under adverse environmental conditions (Paterson et al. 1995; He et al. 2014). Thus, rhizome development is fundamental to the perennial habit in grasses. *Oryza longistaminata*, a wild rice species possessing the AA genome similar to cultivated *Oryza sativa*, is distinguished by its rhizomatous growth habit (Tao and Prapa 2000; Hu et al. 2003). Rhizome development in *O. longistaminata* is influenced by both genetic and environmental factors. Genetic studies have identified over 10 major quantitative trait loci (QTLs) controlling rhizomatousness and more than 50 QTLs affecting rhizome abundance, indicating polygenic regulation through an integrated network (Hu et al. 2003; Fan et al. 2020; Li et al. 2022a, b, Li et al. 2022a, b). Environmental factors influencing rhizome growth include light (Yoshida et al. 2016), sucrose availability (Fan et al. 2017, 2022), nitrogen nutrition (Shibasaki et al. 2021; Kawai et al. 2022), temperature (Wang et al. 2024), and phytohormones, such as auxin, gibberellin and cytokinin (Hu et al. 2011; Shibasaki et al. 2021; Kawai et al. 2022; Yao et al. 2022; Wang et al. 2024; Bessho-Uehara et al. 2025). Despite these insights, the physiological and molecular mechanisms underlying rhizome formation and growth remain inadequately characterized.

The breeding of perennial crops represents a promising strategy for advancing global food security and agricultural sustainability. Through selective introgression of complementary QTLs (e.g., *Rhz2* and *Rhz3*) from *O. longistaminata* into annual *O. sativa* cv. RD23, researchers have developed perennial rice lines featuring shortened rhizomes that enable post-harvest tiller regrowth (Hu et al. 2003; Zhang et al. 2022). RD23 from Thailand is an elite annual *indica* cultivar with broad adaptation (Hu et al. 2003). Subsequent breeding efforts yielded the first perennial rice cultivars, including the *japonica* types PR23 and PR25 (released in China as Yunda25), as well as *indica* cultivars Yunda107 and Yunda109 (Zhang et al. 2022). Remarkably, irrigated perennial rice achieves grain yields comparable to those of annual rice across multiple seasons from a single planting, while reducing labor requirements by 58% and input costs by 49% per regrowth cycle (Samson et al. 2017; Zhang et al. 2017a, b; Huang et al. 2018; Zhang et al. 2019, 2022; Zhang et al. 2021a, b, c). Additionally, continuous perennial rice cultivation enhances soil quality by increasing organic carbon, nitrogen content, pH, and plant-available water capacity (Zhang et al. 2022). These attributes position perennial rice as a transformative crop with potential to improve livelihoods, food security, and soil sustainability (Zhang et al. 2022).

Nitrogen (N) availability profoundly influences rice growth, development, and yield. Tiller number, a key yield determinant, responds strongly to N application: adequate N supply breaks tiller bud dormancy and promotes growth, thereby increasing productive tiller numbers and grain yield (Liu et al. 2022; Yan et al. 2023). Genetic impairments in N acquisition, such as knockout mutations in ammonium transporter genes (*OsAMT1;1*, *OsAMT1;2*, *OsAMT1;3*, *OsAMT3;2*) or nitrate transporter genes (*OsNRT1.1B*, *OsNAR2.2*), reduce N uptake and tillering (Hu et al. 2015; Konishi and Ma 2021; Wu et al. 2024; Hou et al. 2025). Similarly, disruptions in N assimilation genes (e.g., nitrate reductase gene *OsNR2* and glutamine synthetase gene *OsGS1;2* and *OsGS2*) inhibit tiller formation (Ohashi et al. 2014; Gao et al. 2019; Wang et al. 2020). Conversely, manipulation of key transcription factors regulating nitrogen-use efficiency (NUE), such as *OsNGR5*, *OsTCP19*, and *OsGATA8*, alters tiller numbers accordingly (Wu et al. 2020, 2024; Liu et al. 2021a, b). Nitrogen modulates tillering in part through interactions with hormonal pathways (e.g., auxin, gibberellin, cytokinin) in the shoot basal region, though precise mechanisms remain unresolved (Wang et al. 2020; Liu et al. 2022; Yan et al. 2023).

In perennial rice cultivar PR23, N application (0–240 kg ha⁻^1^) progressively increases productive tiller number under field conditions, though regrowth rate is optimized at 120 kg ha⁻^1^, which balances high and stable grain yield (Zhang et al. 2021a, b, c). Nevertheless, the mechanisms through which N regulates tiller regrowth in perennial rice are not fully elucidated. This study identified 1038 DEGs in shoot basal regions of perennial rice following N application, accompanied by reduced IAA levels and attenuated auxin signaling. These changes negatively regulated five key genes involved in N or Mg utilization, potentially enhancing nutrient use efficiency and driving increased tiller regrowth, tiller number, and grain yield.

## Results

### Effect of N Fertilizer on the Tiller Growth and Grain Yield in Perennial Rice

Previous study has shown that moderate N fertilizer application resulted in increased effective tiller number and grain yield in perennial rice PR23 (Zhang et al. 2021a, b, c). Here, another perennial rice cultivar, PR25 was also used to investigate the tiller growth and grain yield under varied N fertilizer urea at the heading stage in the field. At maturity, PR25 produced 438, 459, 476 and 424 tillers/m^2^ and 8.75, 9.59, 11.2 and 9.92 t/ha grain at the 0, 75, 150, 225 kg/ha N fertilizer (Fig. S1), respectively. These results indicate that 150 kg N/ha of heading stage optimally promoted both tiller formation and grain yield in PR25.

### Transcriptome Analysis of Tiller Regrowth

To elucidate the mechanisms underlying N-promoted tiller regrowth in perennial rice, we first examined tiller bud regrowth phenotype following N application at the heading stage in four perennial rice cultivars (PR23, PR25, Yunda107 and Yunda109) and two annual rice cultivars (RD23 and MH63), which showed a similar growth period (Fig. S2). The new small tiller buds were found in the shoot basal regions of perennial rice, PR23, PR25, Yunda107 and Yunda109, at day 7 after 150 kg/ha N fertilizer, with regrowth ratios reaching 80–90% (Fig. S3). In contrast, annual rice RD23, a parent of perennial rice**,** and MH63, a well-known *indica* rice in China, exhibited limited tiller bud regrowth under the same condition, with ratios of only 10–20% (Fig. S3), indicating that N efficiently promoted tiller regrowth in perennial rice.

Subsequently, shoot basal regions were collected from the six selected rice cultivars at two time points, one day before and the seventh day after the application of 150 kg/ha N fertilizer, for RNA sequencing (RNA-seq) analysis. Before N fertilization, raw reads per sample ranged from 36,066,510 to 44,560,520. After quality control and filtering, clean reads accounted for 99.38–99.53% of the total, corresponding to 5.36–6.61 Gb of clean data. In the filtered data, the proportions of bases with quality scores above Q20 and Q30 were 97.54–97.80% and 93.36–93.92%, respectively, with an overall error rate of 0.03–0.07% (Table S1). Principal component analysis (PCA) showed that the first principal component effectively distinguished perennial from annual rice (Fig. S4A), and Pearson correlation analysis indicated good diversity and correlation among the transcriptome datasets (Fig. S4B). Similarly, after N fertilization, raw reads per sample ranged from 36,478,488 to 45,433,444, with clean reads comprising 99.39–99.53% of the total and clean data volumes between 5.41 and 6.74 Gb. The filtered data exhibited Q20 and Q30 base percentages of 97.45–97.92% and 93.15–94.24%, respectively, and an overall error rate of only 0.03–0.04% (Table S2), confirming high base-calling accuracy and reliable data quality. PCA further revealed that the first principal component clearly separated japonica and indica rice, while the second principal component distinguished perennial and annual cultivars (Fig. S4C). Pearson correlation analysis again demonstrated strong dataset diversity and correlation (Fig. S4D). Together, these results provide a foundation for identifying key genes regulating tiller growth in perennial rice in response to N application.

### Identification of Differentially Expressed Genes Associated with Tiller Regrowth

**Fig. 1:**
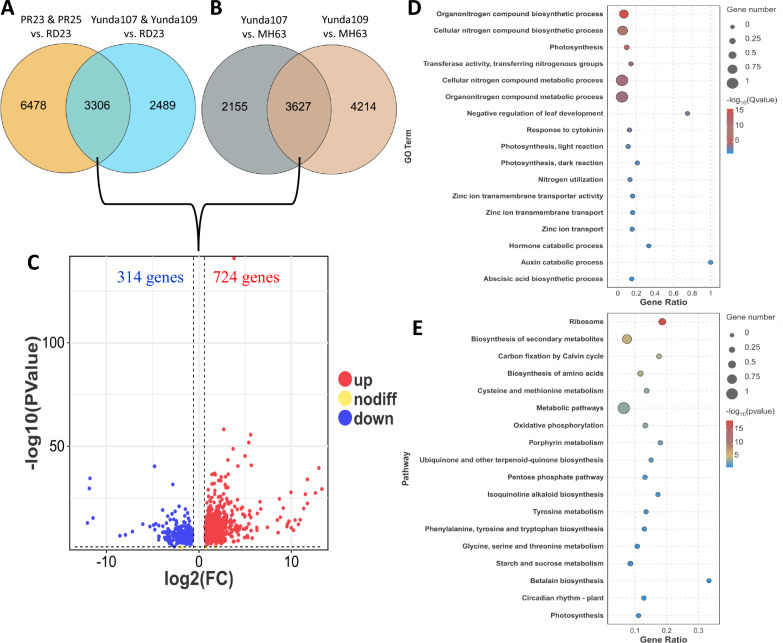
Identification of differentially expressed genes in shoot basal regions between perennial and annual rice after N fertilization. **A** Venn diagram of differentially expressed genes between RD23 and perennial *japonica* rice group (PR23 and PR25) or perennial *indica* rice group (Yunda107 and Yunda109) at day 7 after N fertilizer. **B** Venn diagram of differentially expressed genes between MH63 and Yunda107 or Yunda109. **C** Volcano plot of the 1038 overlapped differentially expressed genes (DEGs) from the Venn diagrams in (**A**) and (**B**). *P* < 0.05, Fold change > 1.5. GO analysis **D** and KEGG analysis **E** of the 1038 overlapped DEGs

Gene expression levels were assessed using FPKM values, and a total of 34,185 genes were successfully mapped by aligning clean reads from both time points. Based on a fold change threshold > 1.5 and a significance level of *P* < 0.05, differentially expressed genes (DEGs) were screened. After N fertilization, comparison between perennial *japonica* rice (PR23 and PR25) or perennial *indica* rice (Yunda107 and Yunda109) with annual rice RD23 (a parent of perennial rice) identified 3,306 DEGs (Fig. 1A), and comparison between perennial *indica* rice (Yunda107 and Yunda109) and annual *indica* rice MH63 identified 3,627 DEGs (Fig. 1B). Integration of these two sets yielded 1,038 DEGs (Table S3), with 314 up-regulated and 724 down-regulated in perennial rice (Fig. 1C, S5C). GO enrichment analysis of these 1,038 DEGs showed significant enrichment in biological processes such as plant hormone signal transduction (e.g., auxin), N metabolism and transport, and photosynthesis (Fig. 1D). KEGG analysis identified 18 significantly enriched pathways, mainly including secondary metabolite biosynthesis, amino acid biosynthesis, oxidative phosphorylation, and photosynthesis (Fig. 1E).

Before N fertilization, a total of 9,549 DEGs were identified via comparison between perennial rice and RD23 (Fig. S5A). Similarly, comparison between Yunda107 or Yunda109 with MH63 yielded 4177 DEGs (Fig. S5A). Integration of these two gene sets resulted in 2102 DEGs (Fig. S5B, Table S4), of which 772 were up-regulated and 1330 were down-regulated in perennial rice (Fig. S5C). GO and KEGG enrichment analysis of these genes revealed significant enrichment in multiple plant hormone-related pathways (Fig. S5D, F), indicating that substantial differences in multiple genes related to plant hormones existed between perennial and annual rice before N fertilization.

To further explore the dynamic changes in DEGs between annual and perennial rice before and after N fertilization, 261 common DEGs were identified (Fig. S5B), which consistently exhibited differential expression independent of N fertilization. GO and KEGG analyses of this gene set revealed significant enrichment in pathways related to chloroplast function, plant hormone regulation, and secondary metabolite biosynthesis (Fig. S5F, G).

### Weighted Gene Co-Expression Network Analysis for Hub Gene Identification

**Fig. 2:**
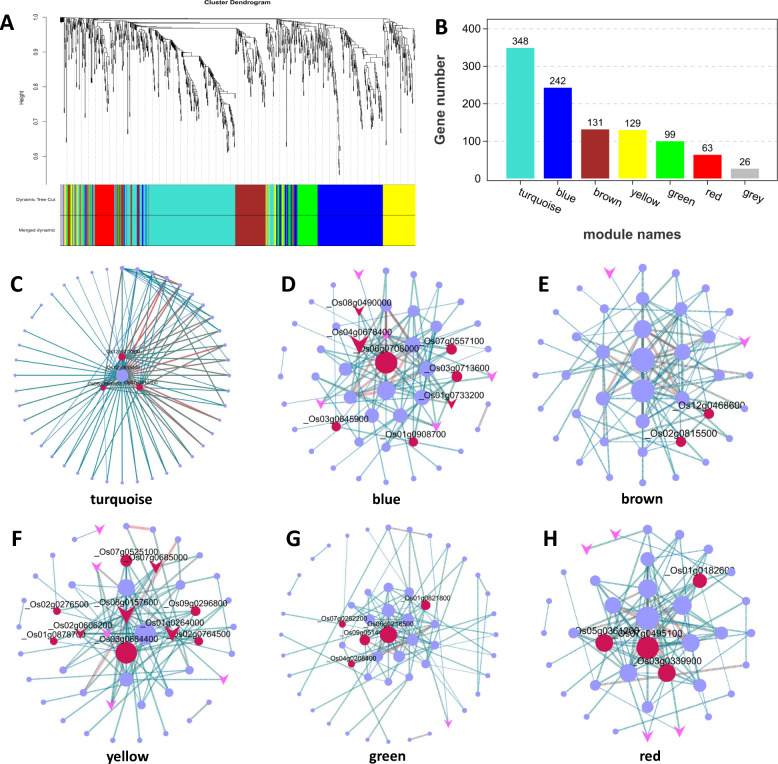
Weighted gene co-expression network analysis of differentially expressed genes between perennial and annual rice after N fertilization. **A** Module hierarchical clustering dendrogram of 1038 DEGs. **B** Gene-module assignment. **C**–**H** Gene regulatory networks in the turquoise, blue, brown, yellow, green, and red modules, respectively. Genes highlighted in red indicate those with previously reported functions or annotated names

To investigate the hub genes governing tiller growth in perennial rice, weighted gene co-expression network analysis (WGCNA) was performed based on tiller bud growth traits (Fig S3) using the 1038 DEGs after N fertilization (Fig. 2A). Hierarchical clustering analysis identified seven modules, turquoise (348 DEGs), blue (242 DEGs), brown (131 DEGs), yellow (129 DEGs), green (99 DEGs), red (63 DEGs), and grey (29 DEGs) (Fig. 2B). Gene regulatory network analysis was further conducted in the former six modules, except of the grey module that contained insufficient genes, leading to identification of a series of core genes (Fig. 2C–H, Table S5).

In the turquoise module (Fig. 2C), two hub genes (*Os12g0277500/OsCpn60α1* and *Os05g0566800/Oscor413-tm1*) are involved in chlorophyll metabolism and drought tolerance (Kim et al. 2013; Zhang et al. 2017a, b), respectively. Besides, the increased expression of the above two genes, nitrate transporter gene *OsNPF7.9*, and amino acid transporter gene *OsAAP1* in perennial rice was confirmed via qRT-PCR (Guan et al. 2022; Pereira et al. 2022) (Fig. S6A). 

The blue module (Fig. 2D) identified two hub genes functionally associated with phytohormone signaling and regulation, including *Os06g0708000/OsMPK12* and *Os03g0645900/OsNCED3* (Koo et al. 2009; Chen et al. 2023). Moreover, genes related to rice growth and development (*Os04g0685600/OsRLS2*) and yield traits (*Os03g0327100/OsNAC17*) were also enriched in this module (Jung et al. 2022; Tu et al. 2015). qRT-PCR confirmed the decreased expression of the above four genes in perennial rice, which may be involved in tiller growth and development. (Fig. S6B).

**Fig. 3:**
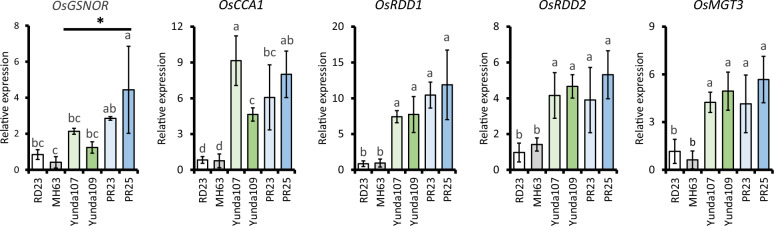
Expression of five hub genes for nutrient utilization in perennial and annual rice. Relative gene expression level of *OsGSNOR* from brown module, *OsCCA1, OsRDD1, OsRDD2* and *OsMGT3* from yellow module in the shoot basal regions of annual rice (RD23 and MH63) and perennial rice (Yunda107, Yunda109, PR23 and PR25) at day 7 after N fertilization. Data are means ± SD (n = 3). Different letters or asterisk represent significant differences (*P* < 0.05) based on Duncan’s test

The brown module (Fig. 2E) featured a hub gene *Os02g0815500/OsGSNOR* with increased expression in perennial rice (Fig. 3), which encodes S-nitrosoglutathione reductase that plays important positive roles in improving N and potassium (K) utilization and biomass under high ammonium conditions (Zhang et al. 2021a, b, c). Additionally, several enriched genes associated with rice tillering and yield, such as *Os01g0802700/OsPIN9*, *Os10g0567400/OsCAO1*, *Os03g0610800/OsSerpin* and *Os12g0197700/OsSGS3b*, were upregulated in perennial rice (Gu et al. 2023; Hou et al. 2021; Jung et al. 2021; Yeu et al. 2007) (Fig. S6C).

Interestingly, the yellow module (Fig. 2F) included multiple core genes associated with nutrient utilization, including *Os08g0157600/OsCCA1/OsNhd1* encoding a transcription factor that plays positively critical roles in regulating N utilization (Li et al. 2022a, b)*, Os01g0264000/OsRDD1* and *Os07g0685000/OsRDD2* encoding transcription factors that play positively key roles in the uptake and utilization of N, phosphorus (P), K and Mg (Iwamoto and Tagiri 2016; Iwamoto et al. 2019)*,* and *Os03g0684400/OsMGT3* encoding a Mg transporter that positively regulates Mg utilization, photosynthetic efficiency and biomass (Li et al. 2020). qRT-PCR confirmed that perennial rice Yunda107, Yunda109, PR23 and PR25 showed 2–10 times higher expression of *OsCCA1, OsRDD1*, *OsRDD2* and *OsMTG3* in the shoot basal regions compared to annual rice RD23 and MH63 (Fig. 3)*.* Additionally, some other hub genes in yellow module (Fig. 2F) were associated with blast resistance (*Os09g0296800* and *Os02g0764500*), auxin transport (*Os07g0525100*), ABA response (*Os02g0606200* and *Os02g0685200*), amino acid transport (*Os01g0878700*), and salt tolerance (*Os02g0276500*) (Ouyang et al. 2011; Yan et al. 2013; Duan et al. 2014; Peng et al. 2014; Liu et al. 2016).

The green module (Fig. 2G) contained several hub genes involved in the development process of pollen viability (*Os09g0514400/OsPFT*), developmental regulation (*Os06g0218500/OsMCM2*) and grain weight (*Os07g0262200/NAL8*) (Chen et al. 2019; Cho et al. 2008; Tao et al. 2021). Furthermore, a gene maintaining panicle development (*Os02g0673100/ALMT7*) was also identified in this module (Heng et al. 2018). These four genes showed an upregulation trend in perennial rice (Fig. S6D).

In the red module (Fig. 2H), a hub gene, *Os01g0182600/OsGI* plays a role in the control of heading date (Itoh & Izawa 2011). Additionally, this module was enriched with genes related to photoperiod and heading date, such as *Os06g0298200/OsBBX19*, *Os03g0284100/OsCCT11* and *Os01g0971800/OsLUX* (Shalmani et al. 2023; Xu et al. 2023; Zhang et al. 2015), all of which exhibited significantly lower expression in perennial rice compared to annual rice (Fig. S6E). The WGCNA results indicate that multi-layered transcriptional networks may regulate tiller regrowth in perennial rice**.**

### Nitrogen Response and Enhanced Nutrient Utilization in Perennial Rice

**Fig. 4:**
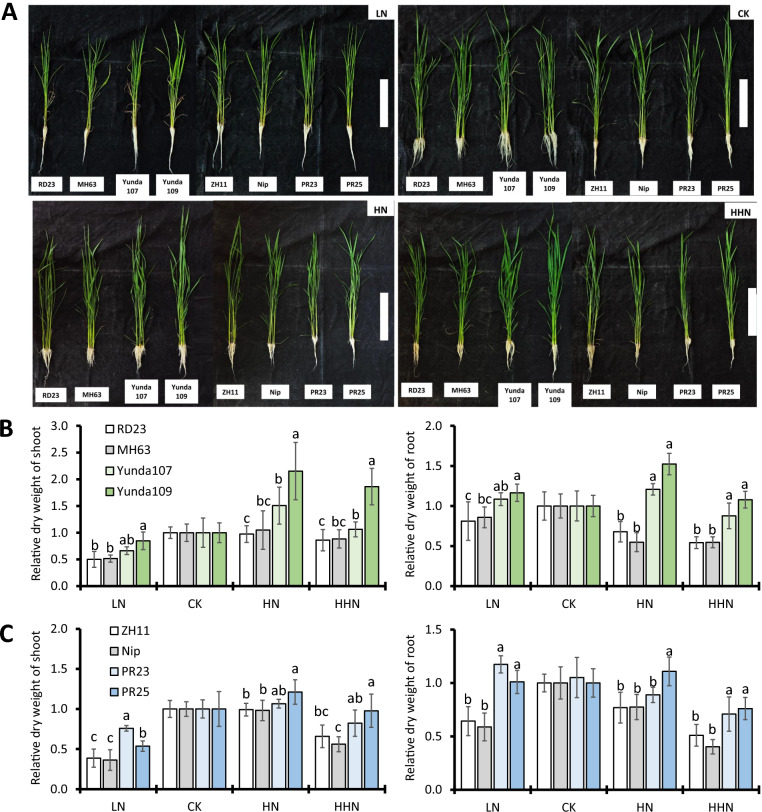
Phenotypic analysis of perennial and annual rice under different N treatments at the vegetative stage. Phenotypes **A** and relative dry weight of shoot and root **B**, **C** in *indica* rice and *japonica* rice among annual rice (RD23, MH63, ZH11 and Nip) and perennial rice (Yunda107, Yunda109, PR23 and PR25). Seedlings were grown under different N treatments (LN: 0.15 mM N, CK: 0.83 mM N, HN: 2.4 mM N, HHN: 4.8 mM N) for three weeks. Data are means ± SD (n = 3). Different letters represent significant differences (*P* < 0.05) based on Duncan’s test. Scale bar: 25 cm

**Fig. 5:**
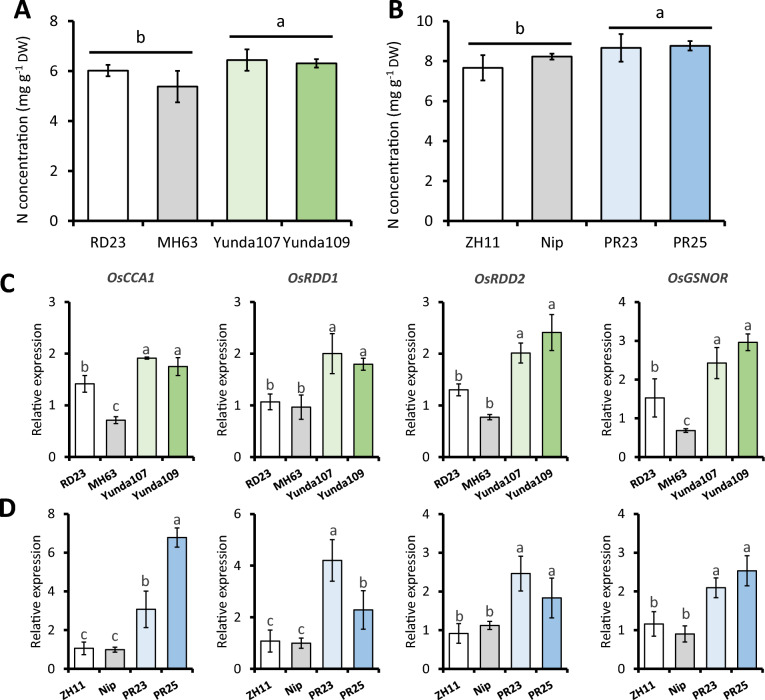
Nitrogen concentration and gene expression for nutrient utilization between perennial and annual rice at the vegetative stage. The N concentration in the seedling (**A**, **B**) and relative expression level of *OsCCA1, OsRDD1, OsRDD* and *OsGSNOR* in the roots (**C**, **D**) were detected in *indica* rice (**A**, **C**) and *japonica* rice (**B**, **D**) among annual rice (RD23, MH63, ZH11 and Nip) and perennial rice (Yunda107, Yunda109, PR23 and PR25) under LN treatment. Data are means ± SD (n = 3). Different letters represent significant differences (*P* < 0.05) based on Duncan’s test

Given that N fertilizer effectively promoted tiller growth and yield in perennial rice (Fig S1, Fig S3), we hypothesize that perennial rice may have enhanced N and nutrient utilization efficiency. To confirm that, rice seedlings were phenotypically analyzed in hydroponic conditions under various N conditions (LN: 0.15 mM, CK: 0.83 mM, HN: 2.4 mM, HHN: 4.8 mM) (Fig. 4). Two annual *japonica* cultivars ZH11 and Nip were used to compare with perennial *japonica* rice PR23 and PR25 for minimizing subspecies variation. Low N partially inhibited rice growth in terms of decreased root or shoot biomass (Fig. 4), especially the shoot of annual rice with inhibition by more than 50%. Perennial *indica* and *japonica* rice showed significantly higher relative shoot and root biomass compared to annual *indica* and *japonica* rice in low N condition (Fig. 4B, C), respectively. Besides, perennial *indica* and *japonica* rice exhibited increased or more stable relative root and shoot biomass in HN and HHN conditions compared to annual *indica* and *japonica* rice (Fig. 4B, C), respectively. Since the LN in hydroponic condition mimicked the field N level (Zhang et al. 2021a, b, c), the N concentration in the seedling was detected. Perennial *indica* and *japonica* rice showed significantly higher N concentration compared to annual *indica* and *japonica* rice (Fig. 5A, B), respectively. These results indicate increased tolerance to low- and high-N and enhanced N utilization in perennial rice.

We performed trait-module association analysis by integrating tiller bud growth trait with the WGCNA modules (Fig. 2, S3, S7). Only the genes in the yellow module exhibited a significant positive correlation with tiller bud growth trait after N fertilization (Fig S7D), suggesting that genes within the yellow module may be the key regulators for tiller bud growth in perennial rice following N fertilization. Consequently, the four hub genes, *OsCCA1*, *OsRDD1* and *OsRDD2* from the yellow module and *OsGSNOR* from the brown module were selected for in-depth investigation. Perennial *indica* and *japonica* rice showed significantly higher expression of the above four genes in the roots than annual *indica* and *japonica* rice (Fig. 5C, D), respectively, suggesting that they may contribute to the increased N utilization in perennial rice*.* Furthermore, the upstream 1 kb regions from the initiator codon ATG of *OsCCA1, OsRDD1*, *OsRDD2, OsGSNOR* and *OsMTG3* were compared between perennial and annual rice. The perennial rice and annual rice did not show distinct haplotypes of the five genes (Fig. S8), indicating that differential expression may arise from variations in upstream regulators or signaling pathways.

### *OsCCA1* Positively Regulates Tiller Bud Growth in Perennial Rice

To confirm the involvement of the hub genes in regulating tiller bud growth in perennial rice, *oscca1* knockout lines in PR25 background, constructed using CRISPR/Cas9 technology, were phenotypically analyzed (Fig. S9). Under LN, CK and HN conditions, LN significantly inhibited tiller bud growth but HN greatly enhanced tiller bud growth compared to CK treatment in WT plants, in terms of ratio and length of tiller bud (Fig. S10A, B). Compared to the separated and unedited WT plants, *oscca1* knockout line did not show visible tiller bud in CK condition and exhibited much fewer and shorter tiller bud in HN condition from 20 to 40 days of treatments, in terms of decreased ratio and length of tiller bud (Fig. S10). These results indicated that *OsCCA1* plays a positive role in tiller bud growth in perennial rice PR25.

To investigate the putative downstream genes of OsCCA1 for tiller bud growth, the putative binding genes of OsCCA1 from previous DAP-seq data (Wei et al. 2022) were integrated with the 1,038 DEGs between perennial and annual rice after N fertilization. 394 genes were identified to be the putative downstream genes of OsCCA1 in regulating tiller bud growth (Fig. S11A), which may be involved in N compound biosynthetic processes, N utilization, developmental regulation, auxin catabolic processes, secondary metabolite biosynthesis, and photosynthetic pathways through GO analysis (Fig. S11B) and KEGG analysis (Fig. S11C).

### Reduced Auxin Level and Its Role on Tiller Growth In Perennial Rice

**Fig. 6:**
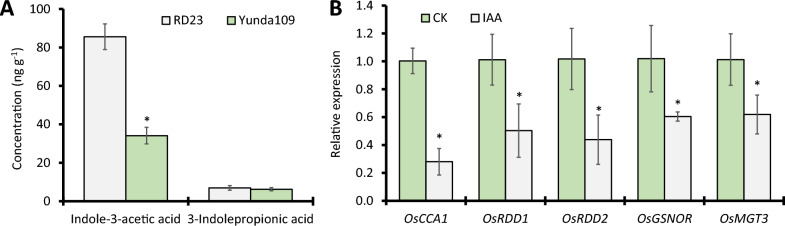
Varied auxin levels in perennial and annual rice and its effect on five key gene expressions. **A** Concentration of auxin-related metabolites in the shoot basal regions of RD23 and Yunda109 at day 7 after N fertilization. **B** Relative expression levels of *OsCCA1*, *OsRDD1*, *OsRDD2*, *OsGSNOR* and *OsMGT3* in the roots of Yunda109 after treatment with 0 or 10 μM IAA for 1 day. Data are means ± SD (n = 6 in A, 4 in B). Asterisk represents significant differences (*P* < 0.05) based on Student's t-test

Since auxin plays a central role in inhibiting axillary bud growth via apical dominance (Yan et al. 2023), and genes involved in the auxin catabolic process showed varied expression in GO analysis (Fig. 1D), the expression of multiple genes involved in auxin metabolism and signal pathway was analyzed. Seven genes for auxin metabolism (Fig. S12A) and six genes for auxin signal pathway (Fig. S12B) were lower expressed in the shoot basal regions in perennial rice (Yunda107, Yunda109, PR23 and PR25) than in annual rice (RD23 and MH63). Furthermore, two common auxins, including indole-3-acetic acid (IAA) and 3-indolepropionic acid (IPA), were measured in *japonica* perennial rice Yunda109 and its parent annual rice RD23. Yunda109 accumulated significantly decreased IAA level by 60% and comparable IPA level than RD23 in the shoot basal regions at day 7 after N fertilizer (Fig. 6A). This reduction in IAA may alleviate apical dominance, facilitating tiller bud regrowth in perennial rice.

Since IAA plays a role in nutrient uptake and utilization (Liu et al. 2022), the effect of IAA on the expression of *OsCCA1, OsRDD1*, *OsRDD2, OsGSNOR* and *OsMTG3* was investigated. Exogenous IAA significantly inhibited the expression of the above five genes, approximately 50%, in the roots of Yunda109 (Fig. 6B). Additionally, exogenous 0.2 or 0.5 μM IAA significantly inhibited axillary bud germination and growth in PR109, with the inhibition intensifying as the IAA concentration increased (Fig. S13). These results suggest that perennial rice likely contained low endogenous IAA levels in shoot basal region, which could promote the expression of genes for nutrient utilization to enhance tiller growth.

## Discussion

**Fig. 7:**
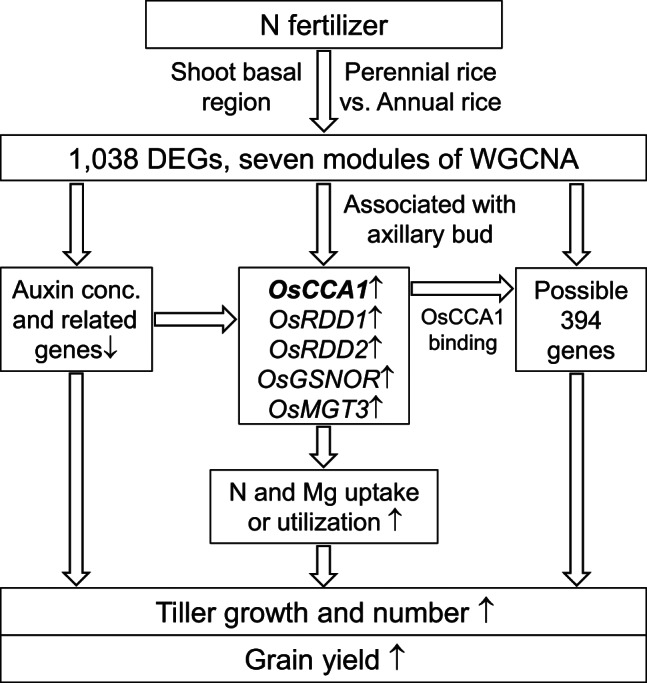
A model to explain N-enhanced tiller regrowth in perennial rice. Perennial rice showed 1,038 DEGs compared to annual rice in the shoot basal regions after N fertilizer application, as well as decreased IAA level that subsequently increased five key genes expression for nutrient utilization, leading to enhanced N and Mg utilization efficiency, resulting in increased tiller regrowth, tiller number and grain yield. OsCCA1 positively regulates tiller bud growth in perennial rice probably via regulating multiple genes of 394 genes

Continuous cropping of perennial rice has been shown to improve soil N retention (Zhang et al. 2022). Application of moderate N fertilizer further enhanced effective tiller number and grain yield in perennial rice PR23 (Zhang et al. 2021a, b, c) and PR25 (Fig. S1), the role of which was similar to that in annual rice (Liu et al. 2022). However, several lines of evidence indicate that perennial rice may possess superior N utilization efficiency (NUE) (Fig. 7). Compared to annual rice, (1) perennial rice showed higher relative biomass under low or high N condition at the vegetative stage (Fig. 4), (2) as well as earlier tiller bud regrowth phenotype after N fertilizer application at the reproductive stage (Fig. S3). (3) Perennial rice accumulated higher N concentration in seedlings than annual rice at the vegetative stage (Fig. 5A, B). (4) Key genes associated with N utilization, including transcription factors OsCCA1, OsRDD1 and OsRDD2, and the S-nitrosoglutathione reductase OsGSNOR (Iwamoto and Tagiri 2016; Iwamoto et al. 2019; Zhang et al. 2021a, b, c; Li et al. 2022a, b), showed significantly elevated expression in the shoot basal regions and roots of perennial rice (Figs. 3, 5C, D). Interestingly, under extremely high N conditions (approximately six times higher), annual rice, particularly *japonica* cultivars, exhibited N toxicity and growth inhibition, whereas perennial rice maintained stable biomass accumulation (Fig. 4), suggesting that perennial rice may possess stronger tolerance to high N stress. The elevated expression of *OsGSNOR*, which confers ammonium tolerance (Zhang et al. 2021a, b, c), could partly explain this phenotypic advantage. On the other hand, to further improve NUE, genetically introduction of the elite genes from other cultivars into perennial rice may be a promising way. For example, introduction of the elite *OsNRT1.1B* from annual *indica* rice 93–11 into PR25 significantly increased effective tillering number (Mi et al. 2024).

N availability is one of the key factors influencing tillering dynamics in rice (Yan et al. 2023). Knockout or inhibited expression of genes for N uptake, homeostasis, metabolism and upstream regulators usually inhibited the tiller growth in annual rice (Ohashi et al. 2014; Hu et al. 2015; Gao et al. 2019; Wang et al. 2020; Konishi and Ma 2021; Wu et al. 2024; Hou et al. 2025). In contrast, overexpression of the key genes for N utilization, such as *OsNGR5*, *OsTCP19* and *OsGATA8*, could enhance tiller growth and grain yield (Wu et al. 2020, 2024; Liu et al. 2021a, b). In perennial rice, the upregulation of *OsRDD1*, *OsRDD2*, *OsCCA1* and *OsGSNOR* likely contributes to improved NUE, thereby promoting tiller regrowth and grain production (Figs. 3, 5, 7). This hypothesis was supported by the fact that knockout of *OsCCA1* inhibited tiller bud growth in perennial rice under different N conditions (Fig. S10). Furthermore, the 394 genes, overlapped by DAP-seq analysis and 1,038 DEGs (Fig. S11), may regulate tiller growth in perennial rice under the control of OsCCA1, although which need to be further researched in future.

Beyond N, OsRDD1 and OsRDD2 also play important roles in the uptake and utilization of P, K and Mg (Iwamoto and Tagiri 2016; Iwamoto et al. 2019). Adequate P and K levels are known to enhance tillering and yield in annual rice (Zhang et al. 2021a, b, c; Jiaying et al. 2022; Yan et al. 2023). Besides, perennial rice showed increased expression of *OsMGT3* (Figs. 3, 5C, D), overexpression of which has been reported to increase photosynthetic efficiency and biomass in rice (Li et al. 2020). Therefore, increased utilization efficiency of P, K and Mg may exist in perennial rice, which likely contributed to the enhanced tiller regrowth in perennial rice (Fig. 7), although this multi-nutrient advantage warrants further investigation.

N modulates tiller growth via complex molecular networks in annual rice (Wang et al. 2020; Yan et al. 2023). For example, recent studies revealed that N-density interactions synergistically regulate root modules involved in N, antioxidant, and energy metabolism, while N-responsive genes likely impact tillering via photosynthetic and C-N metabolic pathways through multi-omics and co-expression network analyses (Sharma et al. 2023; Wang et al. 2025). In this study, the GO and KEGG analysis of the 1,038 DEGs between perennial and annual rice after N fertilizer also revealed multiple biological processes (Fig. 1), such as hormone signaling regulation, primary and secondary metabolism, and photosynthesis. Notably, multiple genes for the metabolism and signaling pathway of auxin were down-regulated in perennial rice compared to annual rice (Fig. S12). The decreased IAA level in the shoot basal regions in perennial rice was also confirmed in this study (Fig. 6A). This aligns with previous reports that N availability affected auxin biosynthesis in annual rice tiller nodes (Xu et al. 2015), and the expression of auxin transporter genes (such as *OsPIN1b*) were inhibited under high N condition in annual rice (Liu et al. 2021a, b), as well as the downregulation of auxin signaling pathway gene (such as *OsIAA12*) and putative weaken polar auxin transport during axillary bud regeneration in perennial rice PR23 (Yao et al. 2022). Furthermore, our results uncovered that exogenous IAA treatment inhibited tiller bud growth in Yunda109 (Figure S13) and IAA inhibited the expression of *OsCCA1*, *OsRDD1*, *OsRDD2, OsGSNOR* and *OsMGT3* (Fig. 6B). Given the well-established role of auxin in apical dominance and axillary bud suppression, and mutants defective in biosynthesis, transport and signaling of auxin showing accelerated outgrowth of axillary buds (Zhao et al. 2001; Xu et al. 2005; Jung et al. 2015; Jia et al. 2024), we propose that reduced auxin level in perennial rice contribute to enhanced tiller regrowth, partly by derepressing genes involved in N and Mg utilization (Fig. 7).

Perennial rice inherits its rhizomatous growth habit from O. *longistaminata*, in which rhizome development is regulated by both N (Shibasaki et al. 2021; Kawai et al. 2022) and auxin (Hu et al. 2011; Shibasaki et al. 2021; Kawai et al. 2022; Yao et al. 2022; Wang et al. 2024). Our study bridges these two regulators within the context of tiller regrowth, positioning perennial rice as a valuable model for exploring the molecular mechanisms underlying perenniality and rhizome development.

## Conclusion

This study elucidates the gene regulatory network and physiological mechanisms mediating N-promoted tiller regrowth in perennial rice (Fig. 7). We demonstrate that N application more effectively induces tiller bud regrowth in perennial rice than in annual rice, associated with 1,038 DEGs in shoot basal tissues. Reduced IAA levels and attenuated auxin signaling in perennial rice alleviate repression of key genes involved in N and Mg utilization, such as *OsRDD1*, *OsRDD2*, *OsCCA1*, *OsGSNOR* and *OsMGT3*, leading to improved nutrient efficiency, increased tillering, and higher grain yield. Moreover, OsCCA1 positively regulates tiller bud growth in perennial rice probably via regulating multiple genes of 394 genes. These insights contribute to a deeper understanding of nutrient-hormone interactions for tiller growth in perennial rice and offer potential strategies for improving N use efficiency in cereal crops.

## Materials and Methods

### Plant Materials and Field Experiment Condition

Four perennial rice cultivars, PR23, PR25/Yunda25, Yunda107 and Yunda109, and four annual *Oryza sativa* rice cultivars RD23, Minghui63/MH63, Nipponbare/Nip and Zhonghua11/ZH11 were used. PR23, PR25, Nip and ZH11 are *japonica*, and Yunda107, Yunda109, RD23 and MH63 are *indica*, which were obtained from School of Agriculture, Yunnan University (Zhang et al. 2022). In paddy field experiment, rice plants were grown at the experimental plots of Yunnan University in Manla Village (N 21.96°, E 100.36°, altitude 1129 m), Xishuangbanna, Yunnan Province, China. The rice plants were subjected to conventional field management until maturity. Different N fertilizer urea, N0 = 0 kg/ha, N75 = 75 kg/ha, N150 = 150 kg/ha, or N225 = 225 kg/ha, was applied to rice in the field at the heading stage.

Under the PR25 background, an *OsCCA1* knockout vector was constructed using the CRISPR/Cas9 system. The target sequence was cloned into the BbsI-linearized pU6 gRNA vector (Mikami et al. 2015), followed by replacement of the gRNA expression cassette into the pZDgRNA-Cas9 ver.2_HPT vector via AscI/PacI digestion. The vector was introduced into PR25 rice callus through Agrobacterium-mediated transformation (Hiei et al. 2006), and T0 generation mutants of *OsCCA1* and WT lines were screened via PCR amplification and sequencing.

### Statistical Analysis of Tiller Regrowth Ratio

The regrowth phenotype and ratio of tiller bud in the shoot basal regions of perennial rice (PR23, PR25, Yunda107 and Yunda109) and annual rice (RD23 and MH63) were recorded at day 7 after N fertilization. Ten seedlings are examined for each variety.

### RNA-Seq Analysis

150 kg/ha urea fertilizer was applied to the rice paddy field at the heading stage. After 7 days, the shoot basal regions (2–3 cm segments) were sample and immediately frozen in liquid nitrogen. Total RNA was extracted and libraries were constructed using the following workflow: mRNA was enriched using mRNA Capture Beads, purified, and fragmented by heat treatment. First-strand cDNA was synthesized from fragmented mRNA using a reverse transcription enzyme mix, followed by second-strand cDNA synthesis, end repair, and A-tailing. Adapters were ligated, and fragments were selected using Hieff NGS® DNA Selection Beads. Finally, PCR amplification was performed to complete library construction. Qualified libraries were sequenced. To ensure data reliability, multi-dimensional quality controls were implemented: RNA integrity and DNA contamination were assessed via agarose gel electrophoresis; RNA purity (OD260/280 and OD260/230 ratios) was measured using a NanoPhotometer spectrophotometer; RNA concentration was quantified precisely with a Qubit 2.0 Fluorometer; and RNA integrity was evaluated using an Agilent 2100 bioanalyzer system. Raw reads were quality-controlled using fastp (Chen et al. 2018) to filter out low-quality data. Analysis was performed using DESeq2 software, with a threshold of *P* < 0.05 and a fold change > 1.5. Data analysis, including Gene Ontology (GO) and Kyoto Encyclopedia of Genes and Genomes (KEGG) analysis were conducted on the Omicsmart platform (https://www.omicsmart.com/).

### Weighted Gene Co-Expression Network Construction And Module Identification

Using the Omicsmart platform (https://www.omicsmart.com/) and R packages (Langfelder & Horvath 2008), a gene co-expression network was constructed via the WGCNA (Weighted gene co-expression cetwork analysis) algorithm. Initially, sample hierarchical clustering was performed to inspect and remove outlier samples. Highly variable genes were then filtered, and network topology analysis was conducted to determine the optimal soft-thresholding power. Modules were identified using dynamic tree cutting with a minimum module size of 50. Modules with high similarity in eigengenes were subsequently merged. Correlation between module eigengenes and the target trait (axillary bud growth in perennial and annual rice at 7 days after fertilization) was calculated to screen for significantly associated modules. Hub genes were finally identified based on module membership (MM) and gene significance (GS).

### Gene Expression Analysis

Total RNA was extracted from shoot basal regions or root using a kit (Omega Bio-Tek, USA). First-strand cDNA was synthesized from 500 ng RNA using a kit (TIANGEN, China). qRT-PCR (QuantStudio7 Flex, Applied Biosystems, USA) was performed with a SYBR Green kit (SuperReal PreMix Plus, TIANGEN, China), using *histone H3* as the internal control. Primer sequences are listed in Table S8.

### Hydroponic Experiment Conditions

Rice seeds were soaked in darkness at 30 °C for 3 days, then transferred to nets floating on 0.5 mM CaCl₂ solution. Seedlings, 5 day-old for the eight cultivars and 2 week-old *oscca1* mutant and its WT lines, were transplanted to ½ Kimura B solution (pH 5.6) with varying nitrogen levels: low nitrogen (LN, 0.15 mM N), control (CK, 0.83 mM N), high nitrogen (HN, 2.4 mM N), or very high nitrogen (HHN, 4.8 mM N). Nutrient solutions were replaced every 2 days. After 21 days, the roots were sampled for gene expression analysis as described above, the plant phenotypes were recorded, and the root and shoot dry weights were measured. Similarly, two-week-old rice seedlings cultured in ½ Kimura B nutrient solution were treated with 10 μM IAA for one day, and then root RNA was extracted to detect the corresponding gene expression levels as described above. After culturing Yunda109 in ½ Kimura B nutrient solution for 3 weeks, the plants were treated with 0.2 and 0.5 μM IAA for 4 weeks to observe the growth phenotype of tiller buds.

### Nitrogen Concentration Measurement

Plant samples were placed in 100 mL digestion tubes with 5 mL concentrated H₂SO₄ for pre-digestion. A stepwise temperature increase digestion method was employed: initial low-temperature heating until white fumes appeared, followed by heating until the solution turned brown-black. After cooling, 300 g·L⁻^1^ H₂O₂ was added dropwise, with each addition followed by 10–20 min of gentle boiling. This process was repeated 2–3 times until the digestate became colorless and transparent, and finally heated for 5–10 min to remove residual H₂O₂. The digestate was diluted to 50 mL, filtered, and reserved for analysis. For Kjeldahl nitrogen determination, 5–25 mL of the test solution was placed in dedicated digestion tubes and quantified via standard acid titration. Blank controls were included throughout the experiment to account for reagent interference.

### Plant Hormones Measurement

Shoot basal regions of RD23 and Yunda109, sampled as described above, were subjected to targeted metabolomics analysis for plant hormones measure. The experiment employed dual-platform LC–MS/MS and GC–MS/MS technologies. The LC–MS/MS system consisted of a Waters Acquity UPLC (BEH C18/HSS T3 column) coupled with an AB SCIEX 5500 QQQ-MS spectrometer, using a water–methanol gradient elution program (8 min) containing 10 mM ammonium formate. The GC–MS/MS system utilized a Thermo Trace1300-ISQ7000 setup with a DB-5 ms column, separating medium- and long-chain fatty acids via an optimized temperature program (140–280 °C). Metabolites were extracted using a methanol–water-formic acid (15:4:1) system containing 0.5% BHT, purified via solid-phase extraction columns, and validated through strict quality controls (QC sample RSD < 10%) to ensure data reliability (Dunn et al., 2011). QC samples were included throughout the experiment to monitor system stability. Quantification was performed using internal standards and calibration curves (R^2^ > 0.99), and metabolites were identified by referencing the NIST 2017 mass spectral database.

### Gene Expression Visualization

Based on the FPKM data obtained from RNA-seq, Z-score normalization was applied, and the TBtools software (Chen et al. 2020) was utilized to generate a heatmap, enabling intuitive visualization and analysis of gene expression patterns.

### Statistical Analysis

Statistical tests were performed using Duncan’s test or Student's t-test. Differences were considered significant at *P* < 0.05.

## Supplementary Information

Supplementary Material 1.

Supplementary Material 2.

## Data Availability

No datasets were generated or analysed during the current study.

## References

[CR1] Bessho-Uehara K, Omori T, Reuscher S, Nagai K, Agata A, Kojima M, Takebayashi Y, Suzuki T, Sakakibara H, Ashikari M, Hobo T (2025) Spatio-Temporal Regulation of Gibberellin Biosynthesis Contributes to Optimal Rhizome Bud Development. Rice 18:39 40410625 10.1186/s12284-025-00798-0 PMC12102016

[CR2] Chen C, Chen H, Zhang Y, Thomas HR, Frank MH, He Y, Xia R (2020) TBtools: an integrative toolkit developed for interactive analyses of big biological data. Mol Plant 13:1194–1202 32585190 10.1016/j.molp.2020.06.009

[CR4] Chen K, Guo T, Li X-M, Yang Y-B, Dong N-Q, Shi C-L, Ye W-W, Shan J-X, Lin H-X (2019) NAL8 encodes a prohibitin that contributes to leaf and spikelet development by regulating mitochondria and chloroplasts stability in rice. BMC Plant Biol 19:395 31510917 10.1186/s12870-019-2007-4 PMC6737680

[CR5] Chen S, Zhou Y, Chen Y, Gu J (2018) fastp: an ultra-fast all-in-one FASTQ preprocessor. Bioinformatics 34:i884–i890 30423086 10.1093/bioinformatics/bty560 PMC6129281

[CR6] Chen Y, Xiang Z, Liu M, Wang S, Zhang L, Cai D, Huang Y, Mao D, Fu J, Chen L (2023) ABA biosynthesis gene OsNCED3 contributes to preharvest sprouting resistance and grain development in rice. Plant Cell Environ 46:1384–1401 36319615 10.1111/pce.14480

[CR7] Cho JH, Kim HB, Kim H-S, Choi S-B (2008) Identification and characterization of a rice MCM2 homologue required for DNA replycation. Biomol Biomed Rep 41:581–586 10.5483/bmbrep.2008.41.8.581 18755073

[CR8] Duan M, Huang P, Yuan X, Chen H, Huang J, Zhang H (2014) CMYB1 encoding a MYB transcriptional activator is involved in abiotic stress and circadian rhythm in Rice. Sci World J 2014:1–9 10.1155/2014/178038 PMC3995101 24977183

[CR83] Dunn W, Broadhurst D, Begley P, Zelena E, Francis-McIntyre S, Anderson N, Brown M, Knowles J, Halsall A, Haselden J, Nicholls A, Wilson I, Kell D, Goodacre R, Human Serum Metabolome Consortium (2011). Procedures for large-scale metabolic profiling of serum and plasma using gas chromatography and liquid chromatography coupled to mass spectrometry. Nat Protoc 6:1060–1083 21720319 10.1038/nprot.2011.335

[CR9] Fan Z, Cai Z, Shan J, Yang J (2017) Letter to the editor: bud position and carbohydrate play a more significant role than light condition in the developmental transition between rhizome buds and aerial shoot buds of *Oryza longistaminata*. Plant Cell Physiol 58:1281–1282 28460115 10.1093/pcp/pcx061

[CR10] Fan Z, Huang G, Fan Y, Yang J (2022) Sucrose Facilitates Rhizome Development of Perennial Rice (*Oryza longistaminata*). Internat J of Molec Sci 23:13396 10.3390/ijms232113396 PMC9654561 36362182

[CR11] Fan Z, Wang K, Rao J, Cai Z, Tao L-Z, Fan Y, Yang J (2020) Interactions Among Multiple Quantitative Trait Loci Underlie Rhizome Development of Perennial Rice. Frontiers Plant Sci 11:591157 10.3389/fpls.2020.591157 PMC7689344 33281851

[CR12] Gao Z, Wang Y, Chen G, Zhang A, Yang S, Shang L, Wang D, Ruan B, Liu C, Jiang H, Dong G, Zhu L, Hu J, Zhang G, Zeng D, Guo L, Xu G, Teng S, Harberd NP, Qian Q (2019) The Indica nitrate reductase gene OsNR2 allele enhances rice yield potential and nitrogen use efficiency. Nat Commun 10:5207 31729387 10.1038/s41467-019-13110-8 PMC6858341

[CR14] Guan Y, Liu DF, Qiu J, Liu ZJ, He YN, Fang ZJ, Gong JM (2022) The nitrate transporter OsNPF7.9 mediates nitrate allocation and the divergent nitrate use efficiency between indica and japonica rice. Plant Physiol 189:215–229 35148397 10.1093/plphys/kiac044 PMC9070802

[CR13] Gu X, Si F, Feng Z, Li S, Liang D, Yang P, Liu J (2023) The OsSGS3-tasiRNA-OsARF3 module orchestrates abiotic-biotic stress response trade-off in rice. Nat Commun 14:4441 37488129 10.1038/s41467-023-40176-2 PMC10366173

[CR16] Heng Y, Wu C, Long Y, Luo S, Ma J, Chen J, Liu J, Zhang H, Ren Y, Wang M, Tan J, Zhu S, Wang J, Lei C, Zhang X, Guo X, Wang H, Cheng Z, Wan J (2018) OsALMT7 maintains panicle size and grain yield in rice by mediating malate transport. Plant Cell 30:889–906 29610210 10.1105/tpc.17.00998 PMC5969278

[CR15] He R, Salvato F, Park J-J, Kim M-J, Nelson W, Balbuena TS, Willer M, Crow JA, May GD, Soderlund CA (2014) A systems-wide comparison of red rice (*Oryza longistaminata*) tissues identifies rhizome specific genes and proteins that are targets for cultivated rice improvement. BMC Plant Biol 14:46 24521476 10.1186/1471-2229-14-46 PMC3933257

[CR17] Hiei Y, Ishida Y, Kasaoka K, Komari T (2006) Improved frequency of transformation in rice and maize by treatment of immature embryos with centrifugation and heat prior to infection with *Agrobacterium tumefaciens*. Plant Cell Tissue Organ Cult 87:233–243

[CR19] Hou L, Chen D, Pan X, Jiang S, Liu J, Li Q, Liu Y, Tong Y, Zhu L, Hu J (2025) 9311 allele of OsNAR2. 2 enhances nitrate transport to improve rice yield and nitrogen use efficiency. Plant Biotechnol J 23:2501–2511 40162885 10.1111/pbi.70073 PMC12205869

[CR20] Hou M, Luo F, Wu D, Zhang X, Lou M, Shen D, Zhang Y (2021) OsPIN9, an auxin efflux carrier, is required for the regulation of rice tiller bud outgrowth by ammonium. New Phytol 229:935–949 32865276 10.1111/nph.16901

[CR24] Huang G, Qin S, Zhang S, Cai X, Wu S, Dao J, Zhang J, Huang L, Harnpichitvitaya D, Wade L, Hu F (2018) Performance, Economics and Potential Impact of Perennial Rice PR23 Relative to Annual Rice Cultivars at Multiple Locations in Yunnan Province of China. Sustainability 10:1086

[CR21] Hu B, Wang W, Ou S, Tang J, Li H, Che R, Zhang Z, Chai X, Wang H, Wang Y, Liang C, Liu L, Piao Z, Deng Q, Deng K, Xu C, Liang Y, Zhang L, Li L, Chu C (2015) Variation in NRT1.1B contributes to nitrate-use divergence between rice subspecies. Nat Genet 47:834–838 26053497 10.1038/ng.3337

[CR22] Hu F, Tao D, Sacks E, Fu B, Xu P, Li J, Yang Y, McNally K, Khush G, Paterson A (2003) Convergent evolution of perenniality in rice and sorghum. Proc Natl Acad Sci U S A 100:4050–4054 12642667 10.1073/pnas.0630531100 PMC153046

[CR23] Hu F, Wang D, Zhao X, Zhang T, Sun H, Zhu L, Zhang F, Li L, Li Q, Tao D (2011) Identification of rhizome-specific genes by genome-wide differential expression analysis in *Oryza longistaminata*. BMC Plant Biol 11:18 21261937 10.1186/1471-2229-11-18 PMC3036607

[CR25] Itoh H, Izawa T (2011) A study of phytohormone biosynthetic gene expression using a circadian clock-related mutant in rice. Plant Signal Behav 6:1932–1936 22101345 10.4161/psb.6.12.18207 PMC3337181

[CR26] Iwamoto M, Tagiri A (2016) MicroRNA-targeted transcription factor gene RDD1 promotes nutrient ion uptake and accumulation in rice. Plant J 85:466–477 26729506 10.1111/tpj.13117

[CR27] Iwamoto M, Tsuchida-Mayama T, Ichikawa H (2019) Different roles of a transcription factor gene RDD2 with close sequence similarity to RDD1 controlling nutrient ion accumulation in rice. Plant Mol Biol Rep 37:327–333

[CR28] Jia L, Dai Y, Peng Z, Cui Z, Zhang X, Li Y, Tian W, He G, Li Y, Sang X (2024) The auxin transporter OsAUX1 regulates tillering in rice (*Oryza sativa*). J Integr Agric 23:1454–1467

[CR29] Jiaying M, Tingting C, Jie L, Weimeng F, Baohua F, Guangyan L, Hubo L, Juncai L, Zhihai W, Longxing T, Guanfu F (2022) Functions of nitrogen, phosphorus and potassium in energy status and their influences on rice growth and development. Rice Sci 29:166–178

[CR30] Jung H, Lee D-K, Choi YD, Kim J-K (2015) OsIAA6, a member of the rice Aux/IAA gene family, is involved in drought tolerance and tiller outgrowth. Plant Sci 236:304–312 26025543 10.1016/j.plantsci.2015.04.018

[CR31] Jung S, Kim T, Shim J, Bang S, Bin Yoon H, Oh S, Kim J (2022) Rice NAC17 transcription factor enhances drought tolerance by modulating lignin accumulation. Plant Sci 323:111404 35914574 10.1016/j.plantsci.2022.111404

[CR32] Jung Y, Lee H, Yu J, Bae S, Cho Y, Kang K (2021) Transcriptomic and physiological analysis of OsCAO1 knockout lines using the CRISPR/Cas9 system in rice. Plant Cell Rep 40:1013–1024 32980909 10.1007/s00299-020-02607-y

[CR33] Kawai M, Tabata R, Ohashi M, Honda H, Kamiya T, Kojima M, Takebayashi Y, Oishi S, Okamoto S, Hachiya T, Sakakibara H (2022) Regulation of ammonium acquisition and use in *Oryza longistaminata* ramets under nitrogen source heterogeneity. Plant Physiol 188:2364–2376 35134987 10.1093/plphys/kiac025 PMC8968255

[CR34] Kim S-R, Yang J II, An G (2013) OsCpn60α1, encoding the plastid chaperonin 60α subunit, is essential for folding of rbcL. Mol Cells 35:402–409 23620301 10.1007/s10059-013-2337-2 PMC3887859

[CR35] Konishi N, Ma JF (2021) Three polarly localized ammonium transporter 1 members are cooperatively responsible for ammonium uptake in rice under low ammonium condition. New Phytol 232:1778–1792 34392543 10.1111/nph.17679

[CR36] Koo SC, Choi MS, Chun HJ, Park HC, Kang CH, Shim SI, Chung J II, Cheong YH, Lee SY, Yun D-J, Chung WS, Cho MJ, Kim MC (2009) Identification and characterization of alternative promoters of the rice MAP kinase gene OsBWMK1. Mol Cells 27:467–474 19390828 10.1007/s10059-009-0062-7

[CR37] Langfelder P, Horvath S (2008) WGCNA: an R package for weighted correlation network analysis. BMC Bioinformatics 9:559 19114008 10.1186/1471-2105-9-559 PMC2631488

[CR38] Li J, Yokosho K, Liu S, Cao HR, Yamaji N, Zhu XG, Liao H, Ma JF, Chen ZC (2020) Diel magnesium fluctuations in chloroplasts contribute to photosynthesis in rice. Nat Plants 6:848–859 32541951 10.1038/s41477-020-0686-3

[CR39] Li K, Zhang S, Tang S, Zhang J, Dong H, Yang S, Qu H, Xuan W, Gu M, Xu G (2022a) The rice transcription factor Nhd1 regulates root growth and nitrogen uptake by activating nitrogen transporters. Plant Physiol 189:1608–1624 35512346 10.1093/plphys/kiac178 PMC9237666

[CR42] Liu F, Zhang L, Luo Y, Xu M, Fan Y, Wang L (2016) Interactions of *Oryza sativa* OsCONTINUOUS VASCULAR RING-LIKE 1 (OsCOLE1) and OsCOLE1-INTERACTING PROTEIN reveal a novel intracellular auxin transport mechanism. New Phytol 212:96–107 27265035 10.1111/nph.14021

[CR43] Liu Q, Wu K, Song W, Zhong N, Wu Y, Fu X (2022) Improving crop nitrogen use efficiency toward sustainable Green Revolution. Annu Rev Plant Biol 73:523–551 35595292 10.1146/annurev-arplant-070121-015752

[CR44] Liu Y, Wang H, Jiang Z, Wang W, Xu R, Wang Q, Zhang Z, Li A, Liang Y, Ou S, Liu X, Cao S, Tong H, Wang Y, Zhou F, Liao H, Hu B, Chu C (2021a) Genomic basis of geographical adaptation to soil nitrogen in rice. Nature 590:600–605 33408412 10.1038/s41586-020-03091-w

[CR45] Liu Z, Su J, Luo X, Meng J, Zhang H, Li P, Sun Y, Song J, Peng X, Yu C (2021b) Nitrogen limits zinc-mediated stimulation of tillering in rice by modifying phytohormone balance under low-temperature stress. Food and Energy Sec 11:e359

[CR41] Li W, Zhang S, Huang G, Huang L, Zhang J, Li Z, Hu F (2022b) A Genetic Network Underlying Rhizome Development in *Oryza longistaminata*. Frontiers Plant Sci 13:966749 10.3389/fpls.2022.866165 PMC9022102 35463392

[CR47] Mikami M, Toki S, Endo M (2015) Comparison of CRISPR/Cas9 expression constructs for efficient targeted mutagenesis in rice. Plant Mol Biol 88:561–572 26188471 10.1007/s11103-015-0342-x PMC4523696

[CR46] Mi S, Li W, Xu Z, Li X, Li X, Zhao S, Zhang Y, Huang G, Yang Q, Shao L. 2024. Effects of Rice Nitrogen Efficient Utilization Gene NRT1.1B on Agronomic Characters in Perennial Japonica Rice Molecular Plant Breeding. 1–11.

[CR48] Ohashi M, Ishiyama K, Kusano M, Fukushima A, Kojima S, Hanada A, Kanno K, Hayakawa T, Seto Y, Kyozuka J, Yamaguchi S, Yamaya T (2014) Lack of cytosolic glutamine synthetase1;2 in vascular tissues of axillary buds causes severe reduction in their outgrowth and disorder of metabolic balance in rice seedlings. Plant J 81:347–356 25429996 10.1111/tpj.12731

[CR49] Ouyang S, He S, Liu P, Zhang W, Zhang J, Chen S (2011) The role of tocopherol cyclase in salt stress tolerance of rice (*Oryza sativa*). Sci China Life Sci 54:181–188 21318489 10.1007/s11427-011-4138-1

[CR50] Paterson AH, Schertz KF, Lin Y-R, Liu S-C, Chang Y-L (1995) The weediness of wild plants: molecular analysis of genes influencing dispersal and persistence of johnsongrass, *Sorghum halepense* (L.) Pers. Proc Natl Acad Sci U S A 92:6127–6131 11607551 10.1073/pnas.92.13.6127 PMC41655

[CR51] Peng B, Kong H, Li Y, Wang L, Zhong M, Sun L, Gao G, Zhang Q, Luo L, Wang G, Xie W, Chen J, Yao W, Peng Y, Lei L, Lian X, Xiao J, Xu C, Li X, He Y (2014) OsAAP6 functions as an important regulator of grain protein content and nutritional quality in rice. Nat Commun 5:4847 25209128 10.1038/ncomms5847 PMC4175581

[CR52] Pereira E, Bucher BC, Santos L, Lerin J, Catarina C, Fernandes M (2022) The amino acid transporter OsAAP1 regulates the fertility of spikelets and the efficient use of N in rice. Plant Soil 480:507–521

[CR53] Samson BK, Voradeth S, Zhang S, Tao D, Xayavong S, Khammone T, Douangboupha K, Sihathep V, Sengxua P, Phimphachanhvongsod V, Bouahom B, Jackson T, Harnpichitvitaya D, Hu F, Wade LJ (2017) Performance and survival of perennial rice derivatives (*Oryza sativa* L./*Oryza longistaminata*) in Lao PDR. Exp Agric 54:592–603

[CR54] Shalmani A, Ullah U, Tai L, Zhang R, Jing X, Muhammad I, Chen K (2023) OsBBX19-OsBTB97/OsBBX11 module regulates spikelet development and yield production in rice. Plant Sci 334:111779 37355232 10.1016/j.plantsci.2023.111779

[CR55] Sharma N, Madan B, Khan M, Sandhu K, Raghuram N (2023) Weighted gene co-expression network analysis of nitrogen (N)-responsive genes and the putative role of G-quadruplexes in N use efficiency (NUE) in rice. Front Plant Sci 14:1135675 37351205 10.3389/fpls.2023.1135675 PMC10282765

[CR56] Shibasaki K, Takebayashi A, Makita N, Kojima M, Takebayashi Y, Kawai M, Hachiya T, Sakakibara H (2021) Nitrogen Nutrition Promotes Rhizome Bud Outgrowth via Regulation of Cytokinin Biosynthesis Genes and an *Oryza longistaminata* Ortholog of FINE CULM 1. Frontiers Plant Sci 12:670101 10.3389/fpls.2021.670101 PMC8120282 33995465

[CR57] Tao D, Prapa S (2000) Preliminary report on transfer traits of vegetative propagation from wild rice species to *Oryza sativa* via distant hybridization and embryo rescue. Agric Nat Resour 34:1–11

[CR58] Tao W, Lijuan L, Zeyu L, Lianguang S, Quan W (2021) Cloning and characterization of protein prenyltransferase alpha subunit in rice. Rice Sci 28:557–566

[CR59] Tu B, Hu L, Chen W, Li T, Hu B, Zheng L, Li S (2015) Disruption of OsEXO70A1 causes irregular vascular bundles and perturbs mineral nutrient assimilation in rice. Sci Rep 5:18609 26691393 10.1038/srep18609 PMC4686888

[CR60] Wang K, Li J, Fan Y, Yang J (2024) Temperature Effect on Rhizome Development in Perennial rice. Rice 17:32 38717687 10.1186/s12284-024-00710-2 PMC11078906

[CR61] Wang R, Qian J, Fang Z, Tang J (2020) Transcriptomic and physiological analyses of rice seedlings under different nitrogen supplies provide insight into the regulation involved in axillary bud outgrowth. BMC Plant Biol 20:197 32380960 10.1186/s12870-020-02409-0 PMC7206722

[CR62] Wang R, Zhu Q, Wang H, Xiong Q (2025) Transcriptome-based analysis of the co-expression network of genes related to nitrogen absorption in rice roots under nitrogen fertilizer and density. Agronomy. 10.3390/agronomy15061429

[CR63] Wei H, Xu H, Su C, Wang X, Wang L (2022) Rice circadian clock associated 1 transcriptionally regulates ABA signaling to confer multiple abiotic stress tolerance. Plant Physiol 190:1057–1073 35512208 10.1093/plphys/kiac196 PMC9516778

[CR64] Wu K, Wang S, Song W, Zhang J, Wang Y, Liu Q, Yu J, Ye Y, Li S, Chen J, Zhao Y, Wang J, Wu X, Wang M, Zhang Y, Liu B, Wu Y, Harberd NP, Fu X (2020) Enhanced sustainable Green Revolution yield via nitrogen-responsive chromatin modulation in rice. Science. 10.1126/science.aaz2046 32029600 10.1126/science.aaz2046

[CR65] Wu W, Dong X, Chen G, Lin Z, Chi W, Tang W, Yu J, Wang S, Jiang X, Liu X, Wu Y, Wang C, Cheng X, Zhang W, Xuan W, Terzaghi W, Ronald PC, Wang H, Wang C, Wan J (2024) The elite haplotype OsGATA8-H coordinates nitrogen uptake and productive tiller formation in rice. Nat Genet 56:1516–1526 38872029 10.1038/s41588-024-01795-7 PMC11250373

[CR66] Xu J, Zha M, Li Y, Ding Y, Chen L, Ding C, Wang S (2015) The interaction between nitrogen availability and auxin, cytokinin, and strigolactone in the control of shoot branching in rice (*Oryza sativa* L.). Plant Cell Rep 34:1647–1662 26024762 10.1007/s00299-015-1815-8

[CR67] Xu M, Zhu L, Shou H, Wu P (2005) A PIN1 family gene, OsPIN1, involved in auxin-dependent adventitious root emergence and tillering in rice. Plant Cell Physiol 46:1674–1681 16085936 10.1093/pcp/pci183

[CR68] Xu P, Zhang Y, Wen X, Yang Q, Liu L, Hao S, Wu W (2023) The clock component OsLUX regulates rice heading through recruiting OsELF3-1 and OsELF4s to repress Hd1 and Ghd7. J Adv Res 48:17–31 35940490 10.1016/j.jare.2022.08.001 PMC10248732

[CR69] Yan L, Lijing X, Junhua L, Shaojun D (2013) Rice B-box zinc finger protein OsBBX25 is involved in the abiotic response. Chin Bull Bot 47:366–378

[CR70] Yan Y, Ding C, Zhang G, Hu J, Zhu L, Zeng D, Qian Q, Ren D (2023) Genetic and environmental control of rice tillering. Crop J 11:1287–1302

[CR71] Yao F, Hu Q, Yu Y, Yang L, Jiao S, Huang G, Zhang S, Hu F, Huang L (2022) Regeneration pattern and genome-wide transcription profile of rhizome axillary buds after perennial rice harvest. Frontiers Plant Sci 13:1071038 10.3389/fpls.2022.1071038 PMC9742242 36518502

[CR72] Yeu S, Park B, Sang W, Choi Y, Kim M, Song J, Seo H (2007) The serine proteinase inhibitor OsSerpin is a potent tillering regulator in rice. J Plant Biol 50:600–604

[CR73] Yoshida A, Terada Y, Toriba T, Kose K, Ashikari M, Kyozuka J (2016) Analysis of rhizome development in **Oryza longistaminat*a*, a wild rice species. Plant Cell Physiol 57:2213–2220 27516415 10.1093/pcp/pcw138

[CR74] Zhang C, Li C, Liu J, Lv Y, Yu C, Li H, Zhao T, Liu B (2017a) The OsABF1 transcription factor improves drought tolerance by activating the transcription of COR413-TM1 in rice. J Exp Bot 68:4695–4707 28981779 10.1093/jxb/erx260 PMC5853872

[CR75] Zhang L, Li Q, Dong H, He Q, Liang L, Tan C, Xing Y (2015) Three CCT domain-containing genes were identified to regulate heading date by candidate gene-based association mapping and transformation in rice. Sci Rep 5:7663 25563494 10.1038/srep07663 PMC4286927

[CR76] Zhang L, Song H, Li B, Wang M, Di D, Lin X, Kronzucker HJ, Shi W, Li G, Cuypers A (2021a) Induction of S-nitrosoglutathione reductase protects root growth from ammonium toxicity by regulating potassium homeostasis in *Arabidopsis* and rice. J Exp Bot 72:4548–4564 33772588 10.1093/jxb/erab140

[CR78] Zhang S, Huang G, Zhang J, Huang L, Cheng M, Wang Z, Zhang Y, Wang C, Zhu P, Yu X, Tao K, Hu J, Yang F, Qi H, Li X, Liu S, Yang R, Long Y, Harnpichitvitaya D, Wade LJ, Hu F (2019) Genotype by environment interactions for performance of perennial rice genotypes (*Oryza sativa* L./*Oryza longistaminata*) relative to annual rice genotypes over regrowth cycles and locations in southern China. Field Crops Res. 10.1016/j.fcr.2019.107556

[CR79] Zhang S, Huang G, Zhang Y, Lv X, Wan K, Liang J, Feng Y, Dao J, Wu S, Zhang L, Yang X, Lian X, Huang L, Shao L, Zhang J, Qin S, Tao D, Crews TE, Sacks EJ, Lyu J, Wade LJ, Hu F (2022) Sustained productivity and agronomic potential of perennial rice. Nat Sustain 6:28–38

[CR77] Zhang S, Hu J, Yang C, Liu H, Yang F, Zhou J, Samson BK, Boualaphanh C, Huang L, Huang G, Zhang J, Huang W, Tao D, Harnpichitvitaya D, Wade LJ, Hu F (2017b) Genotype by environment interactions for grain yield of perennial rice derivatives (**Oryza sativ*a L.**/*Oryza longistamina*ta*) in southern China and Laos. Field Crops Res 207:62–70

[CR80] Zhang T, He X, Chen B, He L, Tang X (2021b) Effects of different potassium (K) fertilizer rates on yield formation and lodging of rice. Phyton 90:815–826

[CR81] Zhang Y, Huang G, Zhang S, Zhang J, Gan S, Cheng M, Hu J, Huang L, Hu F (2021c) An innovated crop management scheme for perennial rice cropping system and its impacts on sustainable rice production. Eur J Agron. 10.1016/j.eja.2020.126186

[CR82] Zhao Y, Christensen SK, Fankhauser C, Cashman JR, Cohen JD, Weigel D, Chory J (2001) A role for flavin monooxygenase-like enzymes in auxin biosynthesis. Science 291:306–309 11209081 10.1126/science.291.5502.306

