# Supplementary material for: Identification of Gene Regulatory Network for Nitrogen-Promoted Tiller Regrowth in Perennial Rice
Source: Rice (N Y). 2026 Apr 28;19:39. doi: 10.1186/s12284-026-00901-z (PMC13269578; doi:10.1186/s12284-026-00901-z)
Supplement: Supplementary file 1 — Supplementary Material 1. [file 12284_2026_901_MOESM1_ESM.pdf]

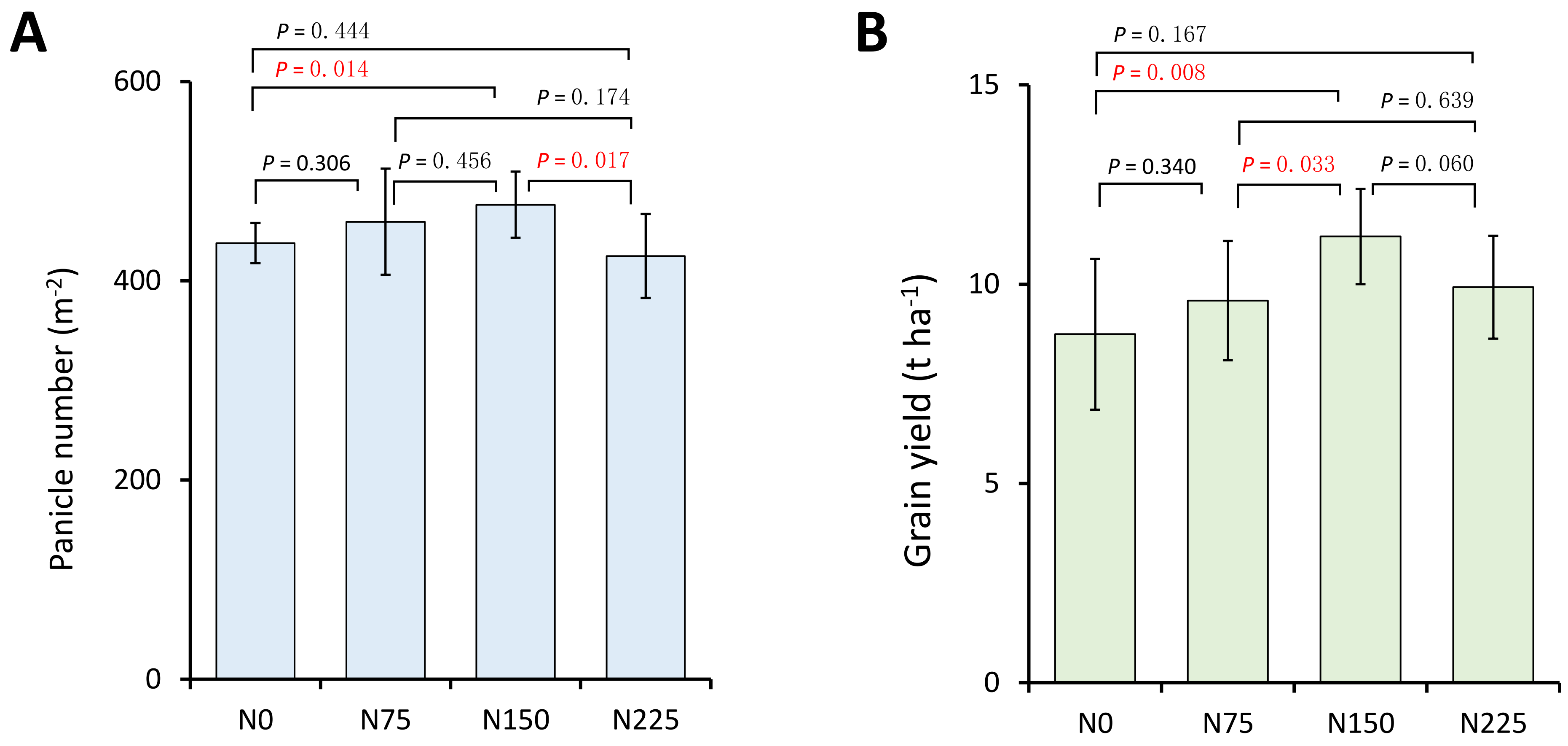

Fig S1. The effect of N fertilizer application on effective panicle number and grain yield in PR25.

The panicle number (A) and grain yield (B) of PR25 under four levels of N fertilizer urea at the heading stage. N0 = 0 kg/ha, N75 = 75 kg/ha, N150 = 150 kg/ha, and N225 = 225 kg/ha. Data are means ± SD (n = 8). Different red numbers represent significant differences ($P < 0.05$) based on Student's t-test.

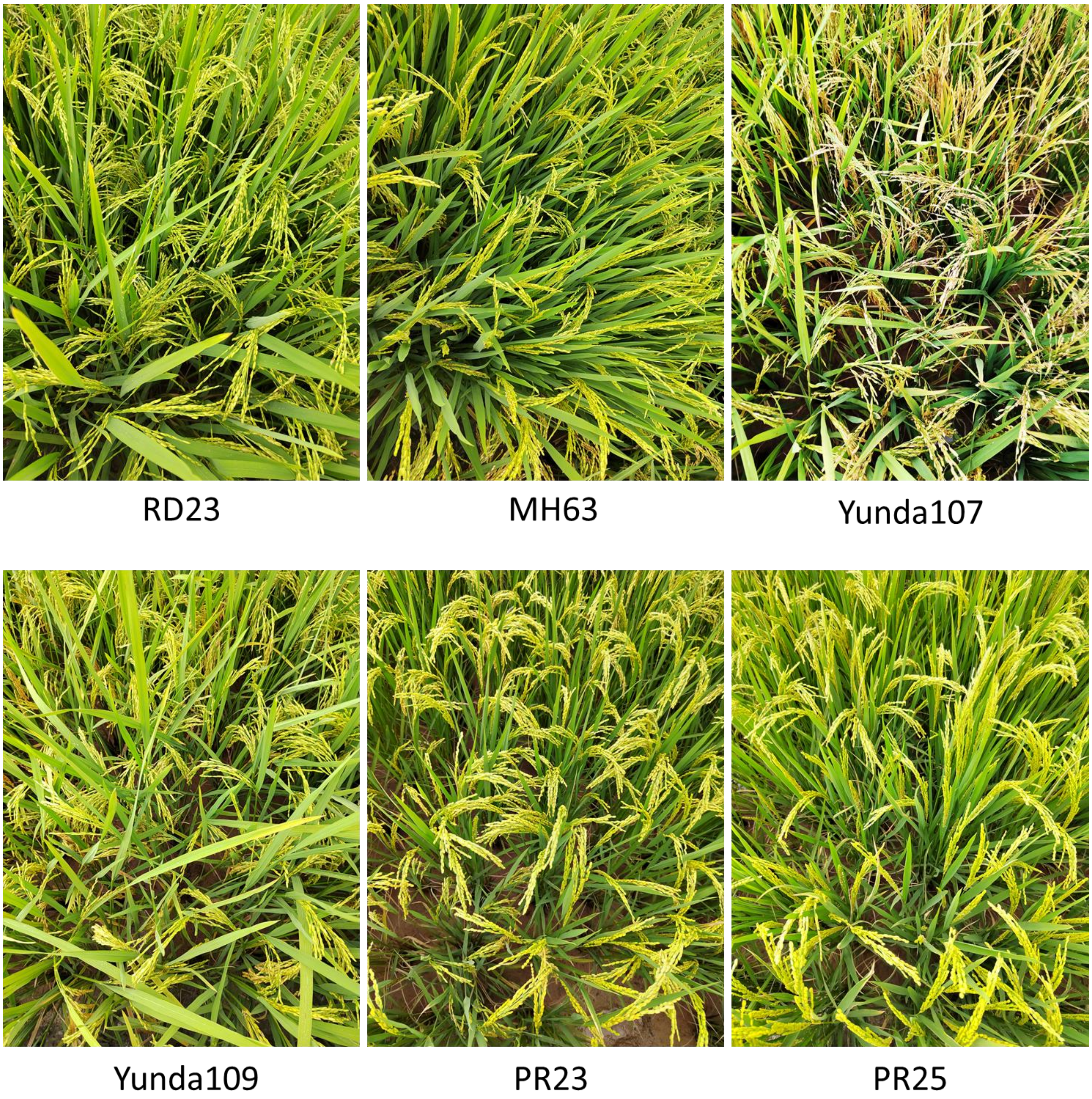

Fig S2. Phenotypes of perennial and annual rice at the reproductive stage. Perennial rice (Yunda 107, Yunda 109, PR23, PR25) and annual rice (RD23, MH63) were grown in the field and the phenotypes were recorded at the reproductive stage.

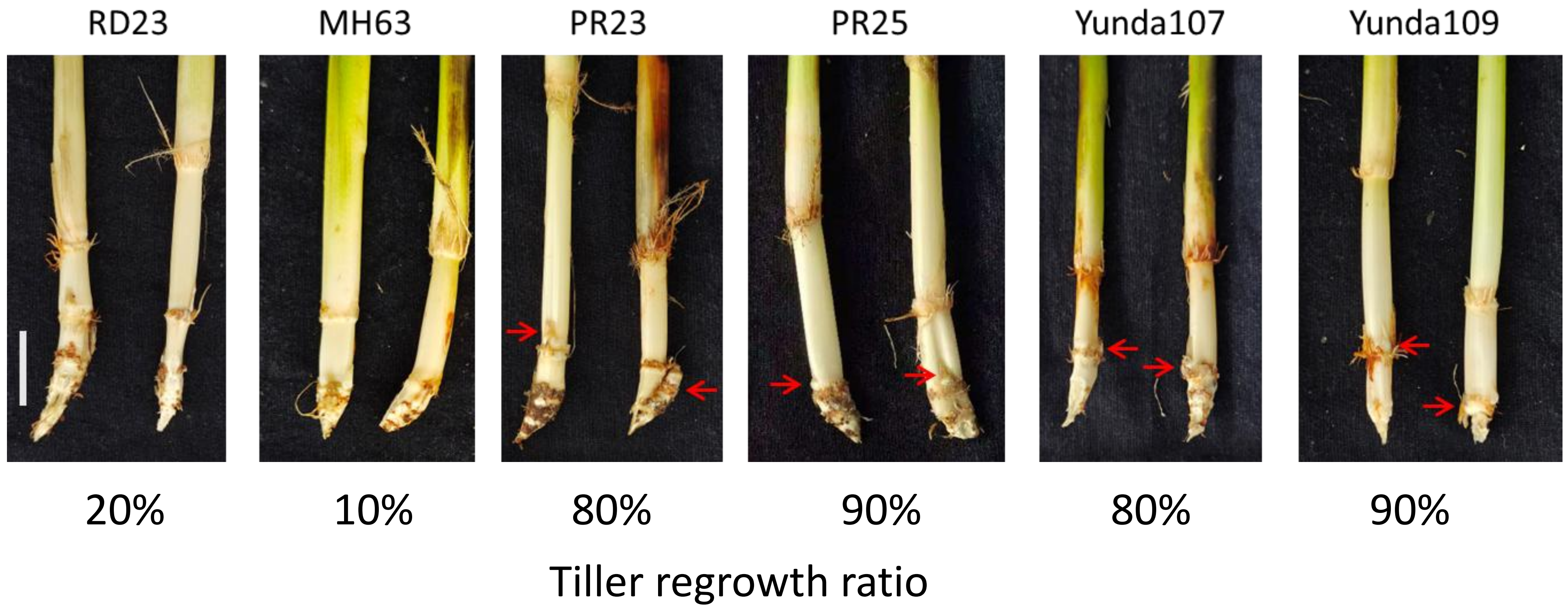

Fig S3. Phenotype of tiller bud regrowth in perennial and annual rice.

The regrowth phenotype and ratio of tiller bud in the shoot basal regions of perennial rice (PR23, PR25, Yunda107 and Yunda109) and annual rice (RD23 and MH63) were shown at day 7 after N fertilization. Ten plants were measured for each variety. Scale bar: 5 cm.

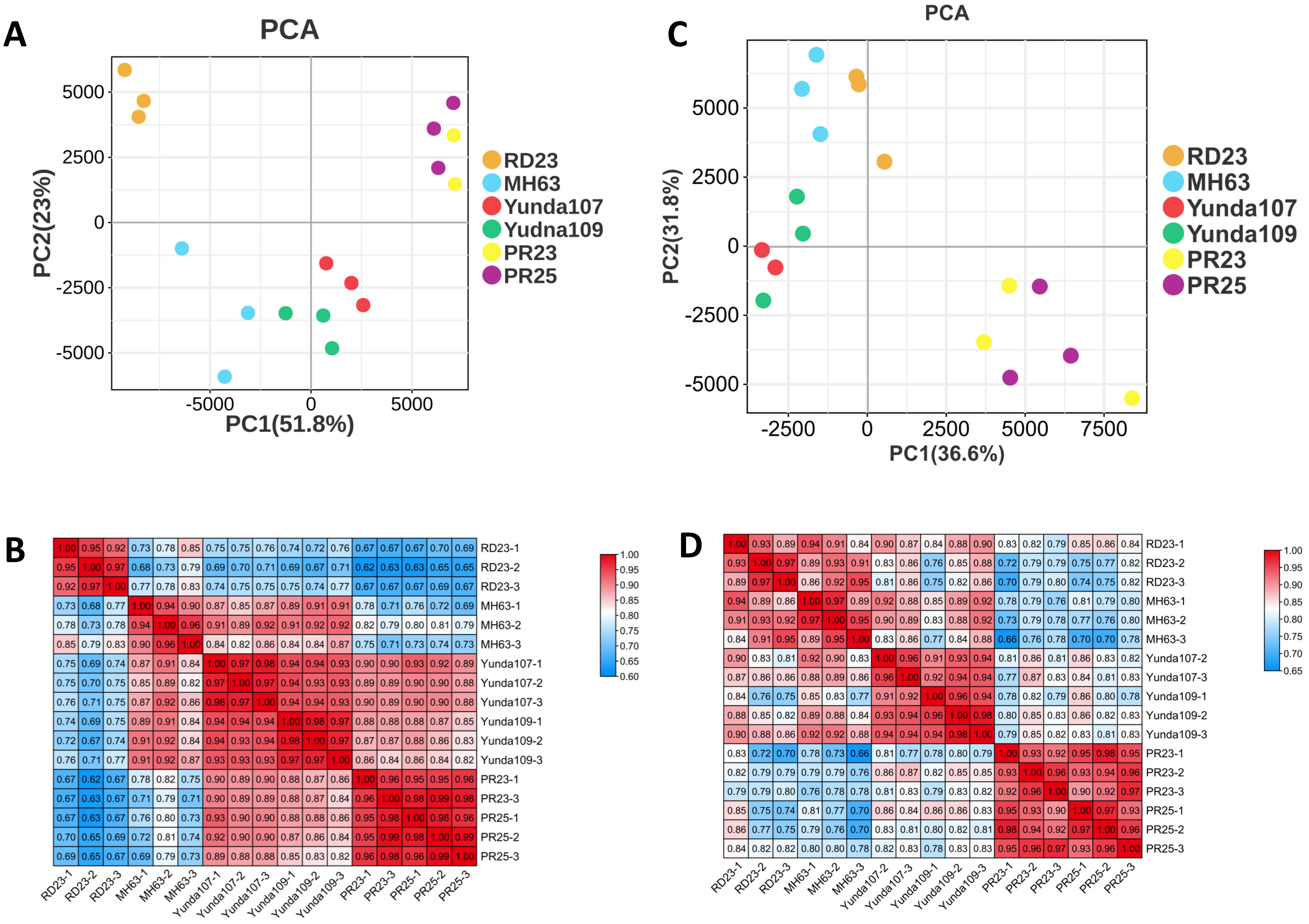

Fig S4. Principal component and correlation analysis of transcriptome data between perennial and annual rice.

Principal component analysis (PCA) (A, C) and Correlation heatmap (B, D) of transcriptomic samples collected at day 1 before fertilization (A, B) and day 7 after fertilization (C, D).

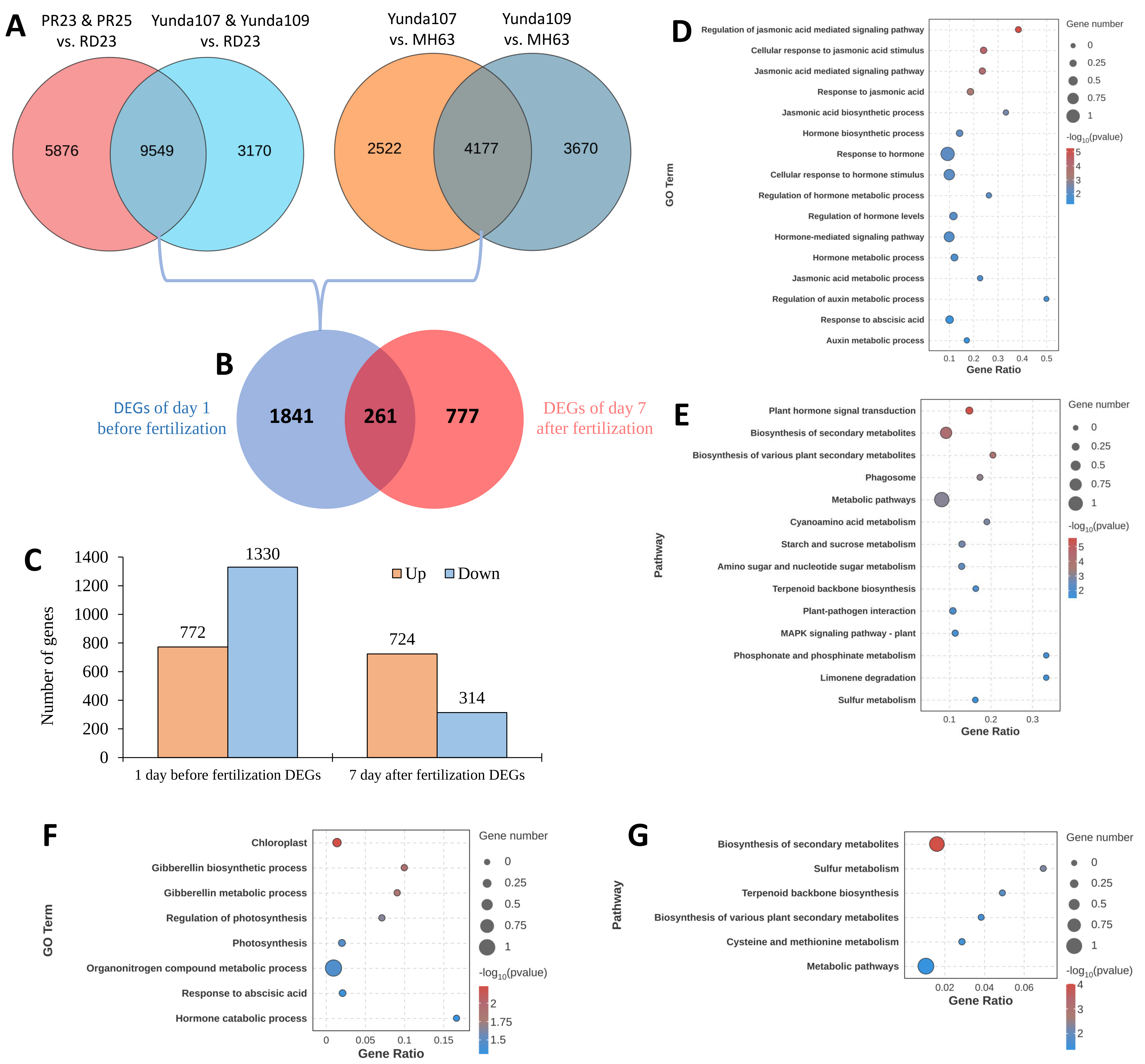

Fig S5. Differentially expressed genes in shoot basal regions between perennial and annual rice.

(A) Venn diagram of DEGs between perennial and annual rice at 1 day before fertilization. (B) Venn diagram (B) and statistics (C) of DEGs in perennial and annual rice at day 1 before fertilization and day 7 after fertilization. GO (D) and KEGG (E) analysis of DEGs in perennial and annual rice at day 1 before fertilization . GO (F) and KEGG (G) analysis of common DEGs in perennial and annual rice at day 1 before fertilization and day 7 after fertilization. $P < 0.05$, Fold change >1.5.

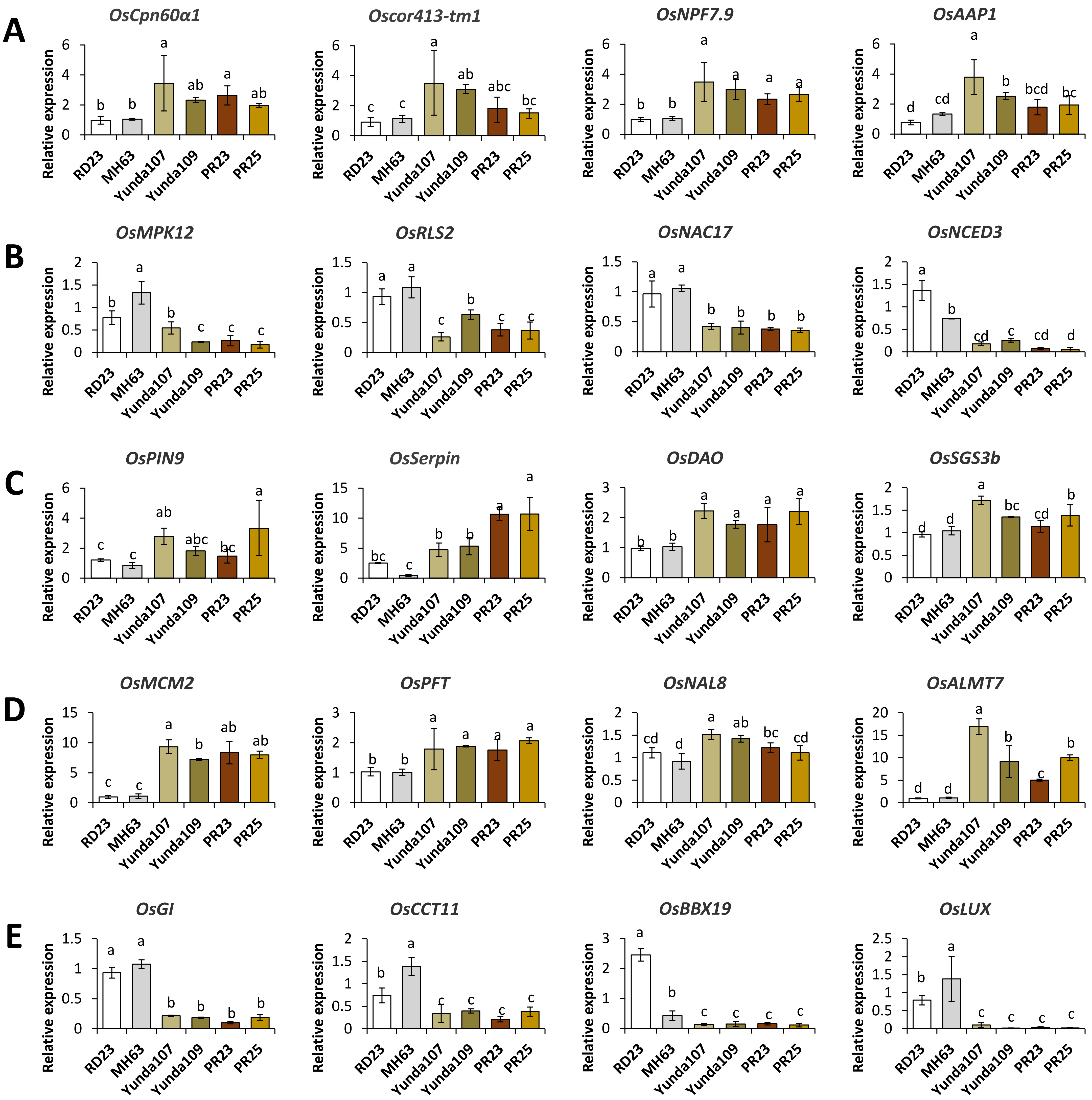

Fig S6. qRT-PCR analysis of genes from distinct modules in the WGCNA after fertilization.

The relative expression of genes in the turquoise (A), blue (B), brown (C), green (D) and red (E) modules in perennial rice (Yunda107, Yunda109, PR23, and PR25) and annual rice (RD23 and MH63). Data are means ± SD (n = 3). Different letters or asterisk represent significant differences ($P < 0.05$) based on Duncan's test.

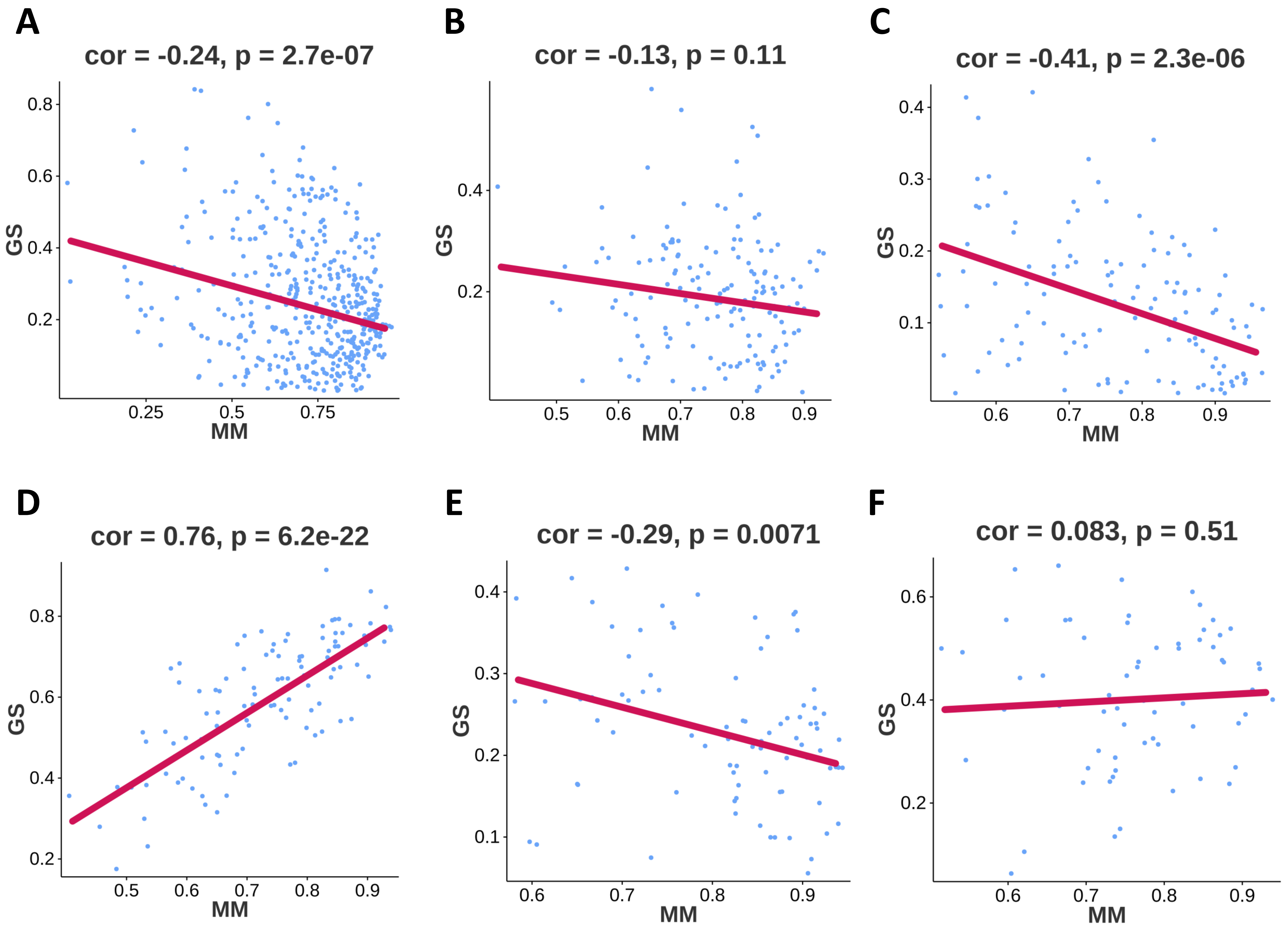

Fig S7. Analysis of the correlation between axillary bud growth traits and WGCNA modules after fertilization.

Correlation analysis between axillary bud growth traits with genes in the turquoise (A), blue (B), brown (C), yellow (D), green (E) and red (F) modules at day 7 after fertilization. *GS* denotes Gene Significance, *MM* indicates Module Membership, *cor* stands for correlation, and $P < 0.05$ signifies statistical significance.

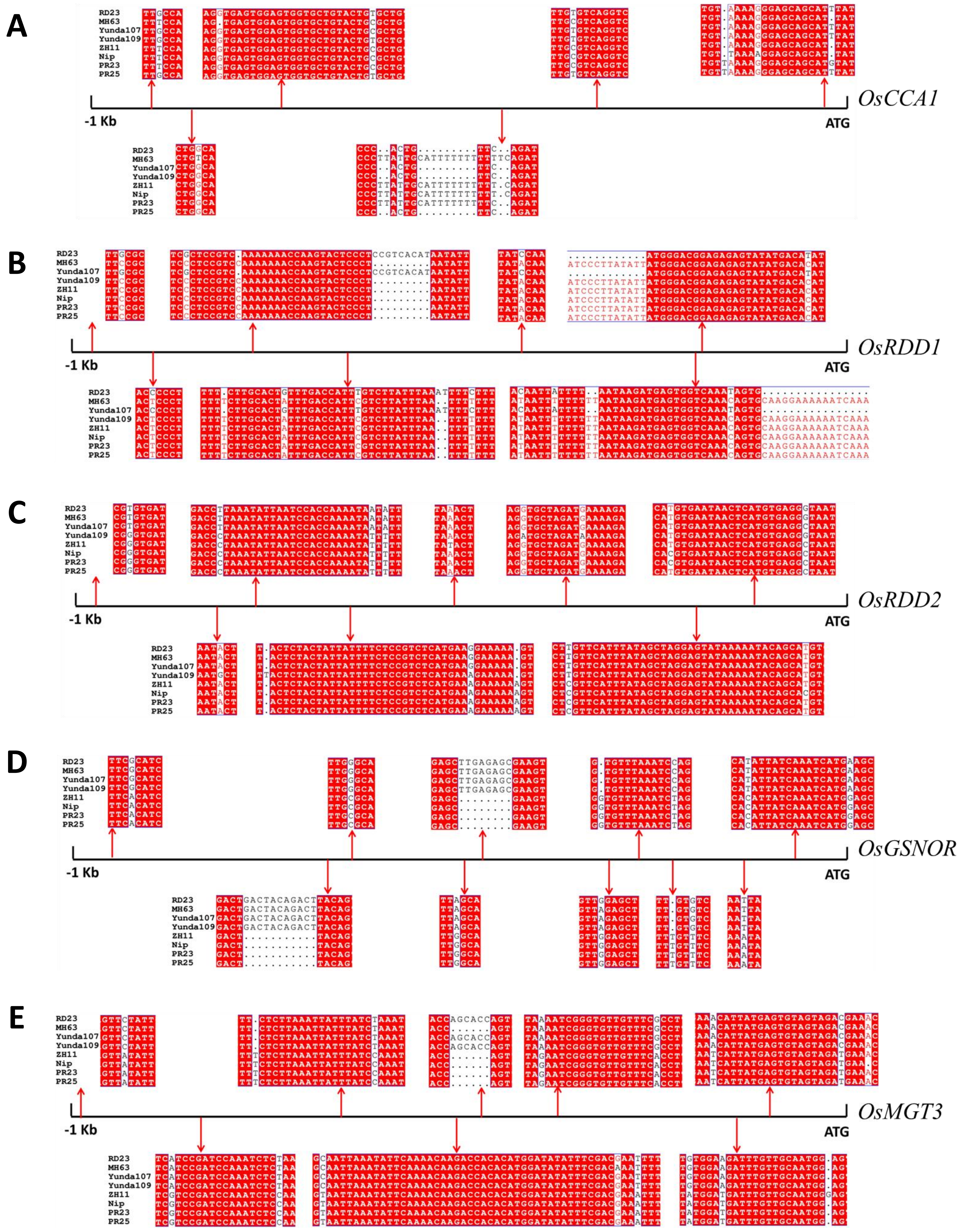

Fig S8. Upstream regions of five key genes in perennial and annual rice.

(A-E) Sequence alignment of 1 kb promoter of *OsCCA1*, *OsRDD1*, *OsRDD2*, *OsGSNOR* and *OsMGT3* genes in perennial rice (Yunda107, Yunda109, PR23 and PR25) and annual rice (RD23, MH63, ZH11 and Nip).

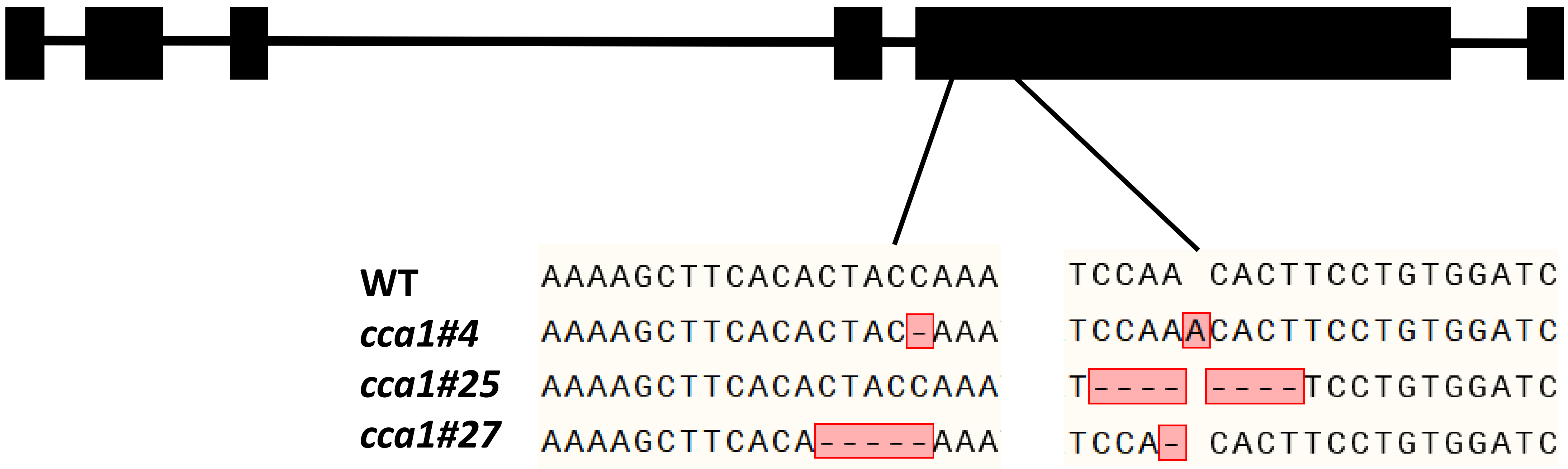

Fig S9. Schematic of CRISPR/Cas9 knockout mutants of *OsCCA1*.

T0 generation of knockout lines of *OsCCA1* and the separated-unedited WT plants were constructed by CRISPR/Cas9 in the PR25 background, and the target positions and sequences are shown.

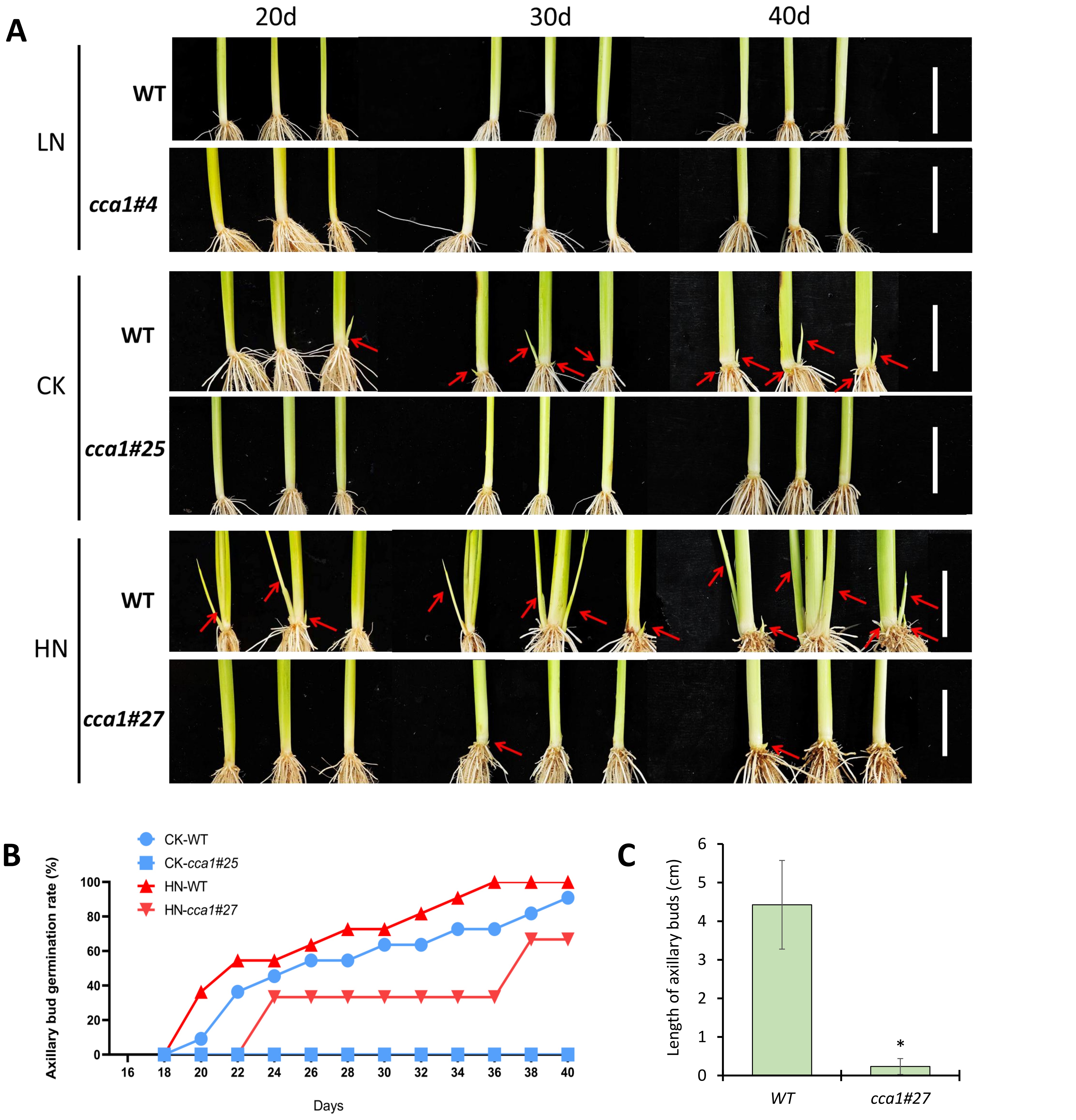

Fig S10. Effect of *OsCCA1* knockout on tiller bud growth under different N conditions.

(A) Tiller bud phenotype of rice under different N conditions at 20, 30 and 40d. (B) Tiller bud germination ratio of rice under CK and HN conditions. (C) Tiller bud length of rice under HN treatment at 40d. Seedlings of *oscca1* mutants and WT were grown under CK condition for 2 weeks, and then treated with different N (LN: 0.15 mM, CK: 0.83 mM, HN: 2.4 mM). Scale bar: 3 cm. Data are means ± SD (n = 4). Asterisk indicates significance (*P* < 0.05, t-test).

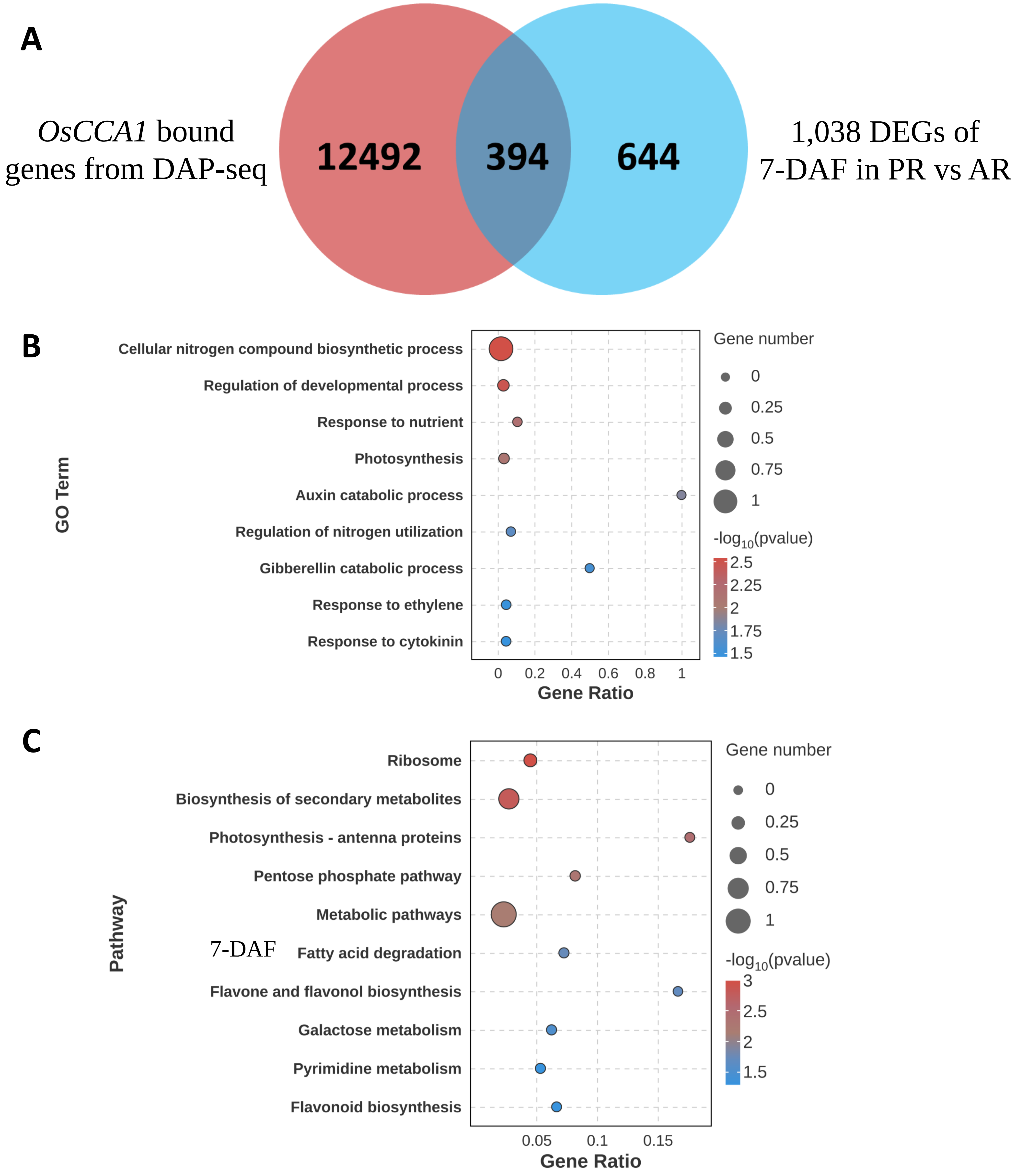

Fig S11. Overlap between OsCCA1-bound genes and 1,038 DEGs of perennial versus annual rice.

(A) Identification of overlapped genes between OsCCA1 binding genes from DAP-seq analysis (Wei et al, 2022) and 1,038 DEGs of 7-DAF in PR vs AR. 7-DAF denotes day 7 after fertilization; AR represents Annual Rice, and PR indicates Perennial Rice. GO (B) and KEGG (C) enrichment analyses of overlapped 394 genes.

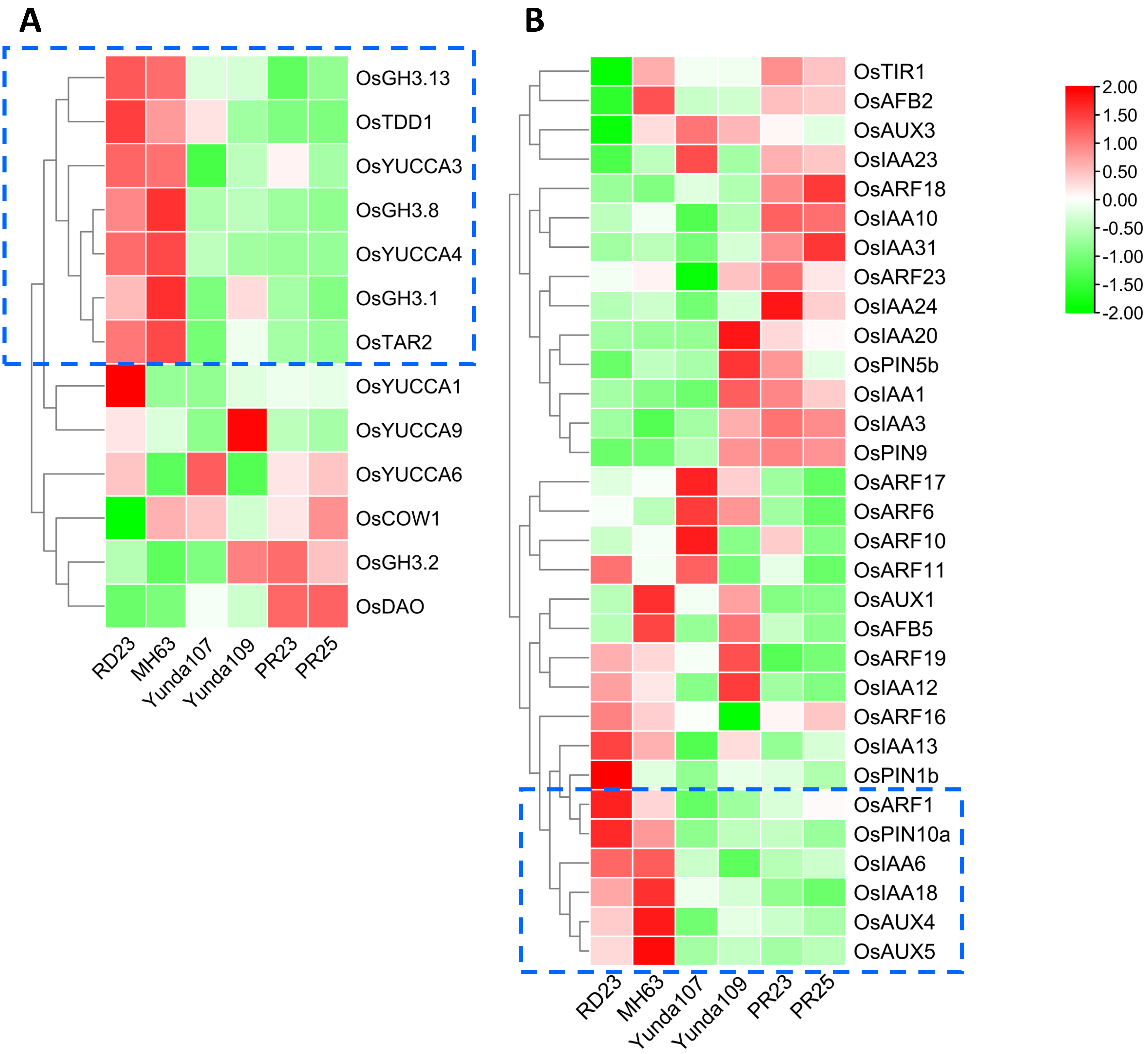

Fig S12. Expression profiles of genes associated with auxin.

The heatmap showed expression patterns of auxin biosynthesis and metabolism-related genes (A) and auxin signaling-related genes (B) in annual rice varieties (RD23 and MH63) and perennial rice varieties (Yunda107, Yunda109, PR23 and PR25) at day 7 after N fertilization. Data were Z-score normalized based on FPKM values of the genes, with red indicating high expression and green indicating low expression.

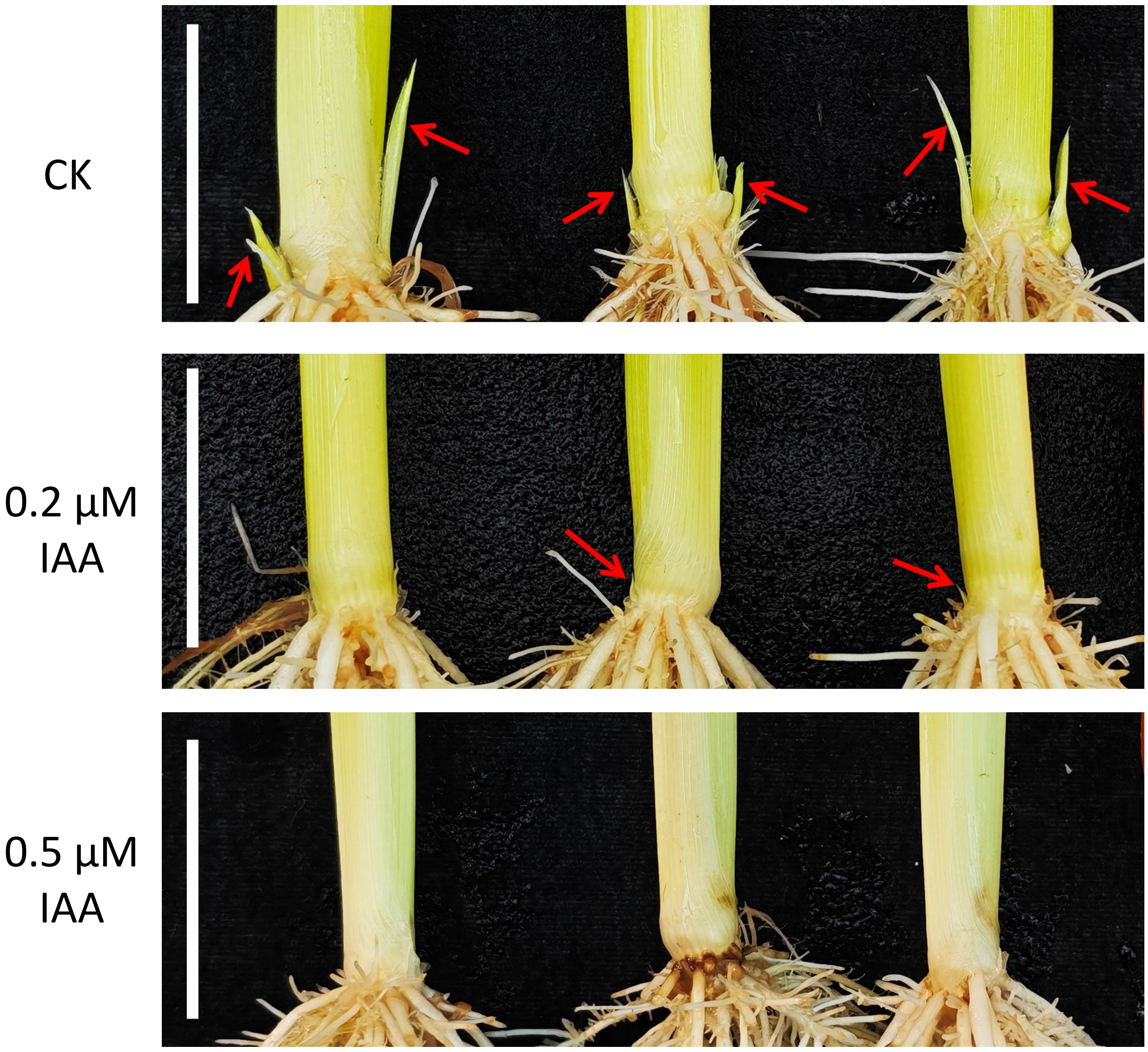

Fig S13. Tiller bud growth of Yunda109 under exogenous IAA treatment. Rice seedlings were grown under CK condition for 3 weeks, and then treated with exogenous 0.2 or 0.5 µM IAA for additional 4 weeks. Scale bar: 3 cm.
